# High-resolution phenotyping identifies NK cell subsets that distinguish healthy children from adults

**DOI:** 10.1371/journal.pone.0181134

**Published:** 2017-08-02

**Authors:** Sanjana Mahapatra, Emily M. Mace, Charles G. Minard, Lisa R. Forbes, Alexander Vargas-Hernandez, Teresa K. Duryea, George Makedonas, Pinaki P. Banerjee, William T. Shearer, Jordan S. Orange

**Affiliations:** 1 Department of Pathology and Immunology, Baylor College of Medicine, Houston, Texas, United States of America; 2 Center for Human Immunobiology, Texas Children’s Hospital, Houston, Texas, United States of America; 3 Department of Pediatrics, Baylor College of Medicine, Houston, Texas, United States of America; 4 Department of Medicine, Baylor College of Medicine, Houston, Texas, United States of America; 5 Residents’ Primary Care Group, Texas Children’s Hospital, Houston, Texas, United States of America; 6 Dan L Duncan Cancer Center, Baylor College of Medicine, Houston, Texas, United States of America; University of Sydney, AUSTRALIA

## Abstract

Natural killer (NK) cells are critical in immune defense against infected, stressed or transformed cells. Their function is regulated by the heterogeneous expression of a wide array of surface receptors that shape its phenotypic diversity. Although NK cells develop in the bone marrow and secondary lymphoid tissues, substantive differentiation is apparent in the peripheral blood including known age-related variation. In order to gain greater insight into phenotypic and functional variation within peripheral blood NK cells across age groups, we used multi-parametric, polyfunctional flow cytometry to interrogate the NK cell variability in 20 healthy adults and 15 5–10, 11–15 and 16–20 year-old children. We found that the normative ranges in both adults and children displayed great inter-individual variation for most markers. While the expression of several receptors did not differ, among those that did, the majority of the differences existed between adults and the three pediatric groups, rather than among children of different ages. Interestingly, we also identified variation in the individual expression of some markers by sex and ethnicity. Combinatorial analysis of NK cell receptors revealed intermediate subsets between the CD56^bright^ and CD56^dim^ NK cells. Furthermore, on examining the NK cell diversity by age, adults were discovered to have the lowest developmental diversity. Thus, our findings identify previously unappreciated NK cell subsets potentially distinguishing children from adults and suggest functional correlates that may have relevance in age-specific host defense.

## Introduction

Natural killer (NK) cells are lymphocytes of the immune system that generally recognize diseased cells without prior antigen sensitization [1]. NK cells arise in the bone marrow and further differentiate in secondary lymphoid tissues via a series of coordinated steps which culminate with acquisition of functional competency [2]. They comprise 5–15% of peripheral lymphocytes and express a wide array of germline-encoded receptors. NK cells are historically identified by their surface expression of CD56 in concert with a lack of expression of CD3 [3]. CD56 can further discriminate two distinct NK cell populations in the peripheral blood based on its level of expression, namely, CD56^bright^ and CD56^dim^ NK cells [4].

Upon detecting a target cell, NK cells utilize a variety of receptor-ligand interactions to establish an immunological synapse (IS) [5]. Adhesion receptors such as integrins (lymphocyte function-associated antigen-1/ LFA-1, macrophage-1 antigen/ Mac-1) function critically in target cell binding. Following stable IS formation, target cells are eliminated via cytotoxicity by the directed release of preformed lytic granules contained within NK cells [6, 7]. In addition to cytotoxicity, NK cells also regulate immunity through their release of cytokines and other soluble factors [1]. These NK cell functions are mediated by integration of signals via engagement of their receptors, which determines the NK cell response in the absence of antigen-restriction [8]. Activation signals that contribute to this balance can be derived from receptors like natural killer group 2 D (NKG2D) and NKp46, which are particularly relevant in pathogen encounters and anti-tumor immunity [9, 10]. Inhibitory signals which counterbalance activation are mediated via receptors that fall into two main families—NKG2 (NKG2A) and killer-cell immunoglobulin-like receptors (KIR) [11]. These recognize MHC class I molecules in order to identify healthy host cells. KIRs are extremely diverse and polymorphic and are crucial in generating diversity in NK cell response to pathogens and as such, have been found to have clinical associations to various diseases [11]. In these cases, an individual's genetic composition and germline expression of KIRs can even be predictive of disease outcome [12–19]. Furthermore, certain NK cell populations have been found to expand in the context of viral infection such as with human cytomegalovirus (hCMV), human immunodeficiency virus (HIV), Epstein-Barr virus, hepatitis C virus and chikungunya virus [20–26]. Thus, NK cell response and a resulting phenotypic distribution is not only defined by hardwired germline-encoded receptors but can also be altered by environmental factors.

While NK cells express a wide variety of surface receptors, the levels of their expression are in some cases defined by the maturity of the NK cell and its developmental stage. NK cell development occurs in discrete stages emanating from the common lymphoid progenitor (CLP) [2]. Acquisition of CD122, the IL-15 receptor β chain, marks the beginning of CLP differentiation towards NK cells since IL-15 is critical for NK cell maturation, differentiation and survival. During NK cell development, NK cells progressively acquire CD161; CD56, CD94/NKG2A, NKp46 and NKG2D; and finally CD16 and KIR. Accordingly, NK cell development occurs in 5 stages whereby stages 4 and 5, the main stages found in peripheral blood, are CD56^bright^ and CD56^dim^ respectively. CD56^bright^ NK cells are characterized by high expression of CD94. CD56^dim^ NK cells acquire KIR and CD16, which marks their functional maturation into fully cytotoxic cells that may become further terminally differentiated and express CD57 [2, 27]. Additionally, CD56^bright^ NK cells effectively produce cytokines in response to cytokine stimulation while CD56^dim^ NK cells are more efficient in producing cytokines on activating receptor engagement [28]. NK cell ability to produce cytokines is also associated with specific developmental stages [29]. While tumor necrosis factor alpha (TNFα) is produced throughout differentiation, the ability to produce interferon gamma (IFNγ) is acquired late, concurrent with CD56 expression and decreased potential to produce interleukins IL-5 and IL-13. While CD56^bright^ and CD56^dim^ NK cells are regarded as distinct stages with disparate functions, several studies have reported the existence of intermediate subsets such as CD56^dim^ CD62L^+^ polyfunctional NK cells, CD94^high^ CD56^dim^ and CD56^bright^ CD16^+^ NK cells [28, 30, 31]. These subsets are also functional intermediaries, which further highlights how unique receptor combinations can delineate NK cell function. Importantly, NK cell function is closely related to its phenotypic diversity [32]. NK cell diversity correlates with experience, either to infections [33] or cytokine stimulation [34]. The emerging theme is that NK cell diversity accrues over the course of our lifetime as a result of rapid response to different pathogen encounters [33].

Despite advances in the field of NK cell biology, the developing immune system of children has not been well studied with regard to NK cells. Two large-scale studies have quantified the distribution of lymphocyte subsets in the peripheral blood of healthy children [35, 36]. These studies have shown that both total NK cell numbers and percentages are not consistent from birth through late adolescence. This variation may be indicative of phenotypic differences in NK cell subsets not only between children and adults but also among children of different ages. Indeed, the expression of certain activating and inhibitory receptors differs between healthy children and adults [37]. However, earlier studies that have looked at NK cells in children have either included a limited number of NK cell receptors or only comprised of children up to 5 years of age [37, 38]. Thus, there is a need for a more comprehensive approach towards establishing age-specific ranges for NK cell markers in children.

While flow cytometry is commonly used for assessing phenotypic diversity, the vast majority of previous studies have not looked at the combinatorial expression of NK cell receptors [39, 40]. One study that evaluated combinatorial NK cell phenotype, used only resting unstimulated NK cells, and thus did not pursue any activation-induced functional capacity within and changes in NK cell subsets [41]. Moreover, these studies have been conducted in adults and normative ranges of combinatorial phenotypes in children have not been established. Using multi-parametric flow cytometry, we contrasted the NK cell phenotype between 45 healthy children and 20 adults. We also distinguished the NK cell repertoire diversity between children and adults. We included 43 distinct NK cell parameters and performed combinatorial analyses. We were able to identify age-related differences in NK cell phenotype, especially between adults and children. In addition, we identified novel NK cell receptor differences likely associated with sex and ethnicity as well as significant differences in NK cell repertoire diversity with age.

## Materials and methods

### Human subjects and cell preparation

This study was approved by the Institutional Review Board for the protection of human subjects of Baylor College of Medicine. A total of 65 (aged 5 to 60 years) generally healthy individuals were recruited at Texas Children’s Hospital (S1 Table). All subjects provided informed written consent. Peripheral blood mononuclear cells (PBMCs) from whole blood were extracted by density centrifugation over GE Healthcare Ficoll-Paque lymphocyte isolation medium (ThermoFisher Scientific) and cryopreserved in filter-sterilized heat-inactivated fetal bovine serum (HI-FBS) (Atlanta Biologicals) containing 10% dimethyl sulfoxide (ThermoFisher Scientific).

Inclusion criteria for healthy individuals included: those who did not receive medication that could impact immunity (i.e. corticosteroids) or who did not have any clinical indication of having immunodeficiency. The majority of our pediatric cohort was recruited from an Allergy and Immunology (A&I) clinic in Houston, Texas, USA. Approximately 40% of those children in our cohort whose medical histories were available had a food allergy (data not shown)—a condition that affects 1 in 13 children [42]. We classified them as healthy owing to the common nature of this condition and the absence of NK cell phenotypic variation in our cohort between individuals with food allergy and those without. Most of the children attending the A&I clinic were doing so as return visits to update progress with previously instituted treatment plans. The most common concerns were food allergy and allergic rhinitis. All other children that were not recruited from the A&I clinic presented for well-child care visits and not ill and without pressing medical concerns. However, one child did have hypothyroidism and another had the relatively common selective IgA deficiency. While there have been some reports of reduced NK cell activity in hypothyroid patients [43], no NK cell abnormality has been seen in selective IgA deficiency [44]. Since neither of these patients were outliers for individual expression of NK cell markers (data not shown), they were therefore not excluded from the study.

### Antibodies

Antibodies were purchased from commercial sources as specified in S2 Table. All antibodies were monoclonal and were titrated for optimal volume. As listed, most antibodies were specific for the respective markers. Some CD158-specific antibodies, however, recognize more than one killer-immunoglobulin like receptor (KIR). In particular, CD158a reacts with KIR2DL1/S1/S3/S5, CD158b reacts with KIR2DL2/L3/S2, and CD158e1 reacts with KIR3DL1.

### PBMC staining and flow cytometry acquisition

Cryopreserved PBMCs were thawed in complete medium [RPMI 1640 (ThermoFisher Scientific) with 10% HI-FBS (Atlanta Biologicals), 1% L-glutamine, 1% penicillin-streptomycin, 1% HEPES buffer, 1% MEM-NEA and 1% sodium pyruvate (ThermoFisher Scientific)]. The cells were resuspended at a concentration of ~3×10^6^ cells/ml. For surface staining without stimulation (Panels 2–4), cells were incubated immediately in 100 μl PBS 2% FBS buffer with antibodies (to a total volume of 200 μl) for 15–20 min in the dark at room temperature. For Panels 1 and 5, the cells were divided into two portions and phorbol 12-myristate 13-acetate (10 ng/ml, Sigma-Aldrich) and ionomycin (1 μg/ml, Sigma-Aldrich) were added to one of these a total volume of 1 ml. Unstimulated cells from activating receptors panel (Panel 1), did not receive any treatment. Intracellular staining of cells for cytokines and effector molecules (Panel 5) required brefeldin A (10 μg/ml, Sigma-Aldrich) and anti-CD107a to be added to both stimulated and unstimulated PBMCs at the onset of incubation. For both Panels 1 and 5, cells were then incubated at 37°C, 5% CO_2_ for 4 hours. At the end of 4 hours, a cocktail of antibodies was added to cells to stain for surface markers (Panels 1 and 5) for 15–20 min. In order to detect intracellular markers (Panel 5), cells were permeabilized using Cytofix/Cytoperm buffer (BD Biosciences) according to manufacturer’s instructions. A cocktail of intracellular antibodies was then added to cells and allowed to incubate for 45–60 min in the dark at room temperature. All cells at the end of staining (Panels 1–5) were washed and fixed in 1X PBS containing 1% paraformaldehyde (Sigma-Aldrich) and stored in the dark at 4°C until acquisition.

For each donor, at least 1000 NK cells were acquired using a modified LSR Fortessa (BD Biosciences) equipped to detect up to 18 fluorescent parameters.

### Data analysis

Data analysis was performed using Kaluza (Version 1.2, Beckman Coulter). The scale on Kaluza has a maximum limit of 10^3^, which is 2 log decades lower than the scale on LSR Fortessa (up to 10^5^). Therefore, absolute MFI values reported in this study are lower than those during sample acquisition. The conversion factor between the two is approximately LSR Fortessa MFI = Kaluza MFI x 256 [45]. Gating strategy has been depicted in S1 Fig. Treeplot analysis was performed on Kaluza to determine simultaneous expression of 10 markers (including CD45 and CD56) by creating donor-specific treeplots for each panel of antibodies. Spanning tree-progression analysis of density-normalized events (SPADE) was performed using Cytobank, Inc. as described in [46]. Briefly, SPADE analysis was run on the combined NK cells of 5–10 y.o., 11–15 y.o., 16–20 y.o. and adults by concatenating FCS files from multiple donors and gating on NK cells. Different regions of the SPADE plots were then defined by sequentially examining intensity of each marker in the panel. In addition to the relative intensities of each marker within a plot, corresponding fluorescence minus one (FMO) plots were used to determine presence or absence of a particular marker within that subset at the node of interest.

### Diversity quantification

The inverse Simpson index was calculated using the equation as described in [41]

$$\text{D}=\frac{1}{\sum_{\text{i}=1}^{\text{S}}{\text{p}}_{\text{i}}{}^{2}}$$

where S is the total number of species and p_i_ is the proportional abundance of the i^th^ species.

### Statistical analysis

All statistical analysis was performed using Prism (Version 6, GraphPad Software, Inc.), STATA (Version 14, Stata Corp) and SAS (Version 9.4). For markers KIR2DS4 (APC), KLRG1 (APC-Vio770), CD11b (APC-Alexa Fluor 750) and CD122 (PE), 16 adults were analyzed. Comparisons across 4 groups were made using a non-parametric test (Kruskal-Wallis) followed by post-hoc comparison by Dunn test with Bonferroni adjustment. A Mann-Whitney test was used for comparisons across 2 groups. For combinatorial subset statistical analysis, the most common subsets were identified as those with mean percentages at a minimum threshold of 3%. Subsets were compared between or within cohorts if the mean percent gated was at least 3% in a cohort. A general linear mixed model was used to compare the mean percent gated of common subsets between adult and child cohorts. The model included fixed effects for subset, cohort, and the subset-cohort interaction term. The matrix of correlated residuals assumed an unstructured format. The model was used to estimate the mean difference in percent gated between adults and children for each subset. P-values were adjusted for multiple comparisons using Holm’s step-down Bonferroni method, and statistical significance was assessed at the 0.05 level. A separate model was used for each panel-cohort comparison.

## Results

### Normative ranges of NK cell markers in both adults and children display great inter-individual variation and post-stimulation changes

The frequency of NK cells in peripheral blood varies with age, with the highest numbers at birth and again in adolescence, followed by decline in adulthood [35, 36]. Given this age-related variation in NK cells, we sought to establish normative ranges for NK cell subsets in healthy adults and children using multi-dimensional flow cytometry. Our cohort consisted of 20 adults above 21 years of age (22–60 years), and 45 children which were subdivided into three 5-year age groupings—5 to 10 year-olds, 11 to 15 year-olds and 16 to 20 year-olds, which generally correspond to pre-pubertal, pubertal and post-pubertal stages of childhood respectively (S1 Table). NK cell effector functions are regulated by both activating and inhibitory receptors, with contributions from a variety of ancillary and adhesion receptors, all acquired through stages of differentiation and maturation [1, 47]. Therefore, we designed 5 multi-color flow cytometry panels to interrogate the breadth of the NK cell repertoire (Table 1). All panels include CD3, CD45 and CD56 to facilitate positive identification of NK cells (CD45^+^CD3^-^CD56^+^).

**Table 1:** NK cell phenotyping panels.

Panel 1	Panel 2	Panel 3	Panel 4	Panel 5
Activating receptors	Adhesion/ stimulatory receptors	Inhibitory receptors	Developmental markers	Cytokines and effector molecules
**CD45**	**CD45**	**CD45**	**CD45**	**CD45**
**CD56**	**CD56**	**CD56**	**CD56**	**CD56**
**CD3**	**CD3**	**CD3**	**CD3**	**CD3**
CD8	CD16	CD158a/ KIR2DL1	IL-15Rα	Perforin D48
DNAM-1	CD8	CD158b/ KIR2DL2-3	CD117/ c-kit	Perforin δG9
NKG2D	CD2	CD158e/ KIR3DL1	CD127	Granzyme B
NKp30/ NCR3	CD244/ 2B4	KIR2DS4	CD27	CD107a/ LAMP-1
NKp44/ NCR2	CD18	NKG2A	CD62L	TNFα
NKp46/ NCR1	CD11a (LFA-1)	NKG2C	CD94	IFNγ
CD69	CD11b (Mac-1, CR3)	CD94	CD122	IL-5
CD25	CD11c (CR4)	KLRG1	CD16	IL-13
	CD54/ ICAM-1		CD11b	IL-10
	CD28		CD57	

Panel 1 primarily includes activating receptors. NKp30, NKp44 and NKp46 are natural cytotoxicity receptors [10]. NKG2D recognizes stress-induced molecules [48]. CD25 is the IL-2 receptor alpha chain and is upregulated on activated cells, as is CD69 [49, 50]. CD8 on NK cells is an α/α homodimer and both CD8 and DNAM-1 are co-stimulatory receptors [51, 52]. Panel 2 includes adhesion and other stimulatory receptors. On NK cells, the integrin β2 subunit (CD18) primarily pairs with three alpha chains–αL (CD11a), αM (CD11b) and αx (CD11c) [53]. Other notable NK cell receptors that mediate target cell recognition and binding include 2B4 (CD244), CD2, CD28, CD8, CD16 and CD54 (ICAM-1) [54–57]. Panel 3 mostly comprises inhibitory receptors belonging to the killer immunoglobulin-like receptor (KIR) family [58]. Killer cell lectin-like receptor G1 (KLRG1) is another inhibitory receptor found on NK cells that recognizes cadherins on healthy tissues [59]. We also juxtaposed certain activating receptors in Panel 3 as, although NKG2C is activating, both NKG2C and the inhibitory NKG2A form obligate heterodimers with CD94 [60, 61]. Similarly, KIR2DS4 is activating but belongs to the KIR family that also includes CD158a, CD158b and CD158e [11]. Panel 4 consists of major NK cell developmental markers. NK cell development occurs progressively, with CD56^bright^ NK cells thought to be direct precursors of CD56^dim^ NK cells, the two final stages found in the peripheral blood [4, 62]. Generally, CD56^dim^ NK cells constitute 90% of total NK cells while CD56^bright^ subsets comprise the remaining 10% [4, 62]. Distinguishing markers that are acquired during NK cell linear differentiation are CD62L, CD27, CD127, CD117 and CD94 [2, 27]. These are more highly expressed on the CD56^bright^ NK cells, unlike CD11b, CD16, CD122 and CD57, whereby CD57 expression represents terminally differentiated cells. Finally, Panel 5 includes NK cell functional mediators. NK cells degranulate to secrete specialized lysosomes (lytic granules) containing perforin and granzyme B in order to lyse target cells. We have two different perforin clones in our panel. δG9 recognizes lytic granule-associated perforin while D48 also recognizes newly synthesized perforin, which can traffic via an alternate granule-independent pathway [63]. Additionally, this panel includes CD107a, which is a marker of degranulation [64]. NK cells produce both type 1 (NK1) cytokines such as IFNγ and type 2 (NK2) cytokines like IL-5 and IL-13 [29, 65]. Other NK cell cytokines include the inflammatory TNFα and the immunoregulatory IL-10 [66]. Thus, our panels comprise an array of relevant markers that address distinct aspects of NK cell biology and function.

**Fig 1:**
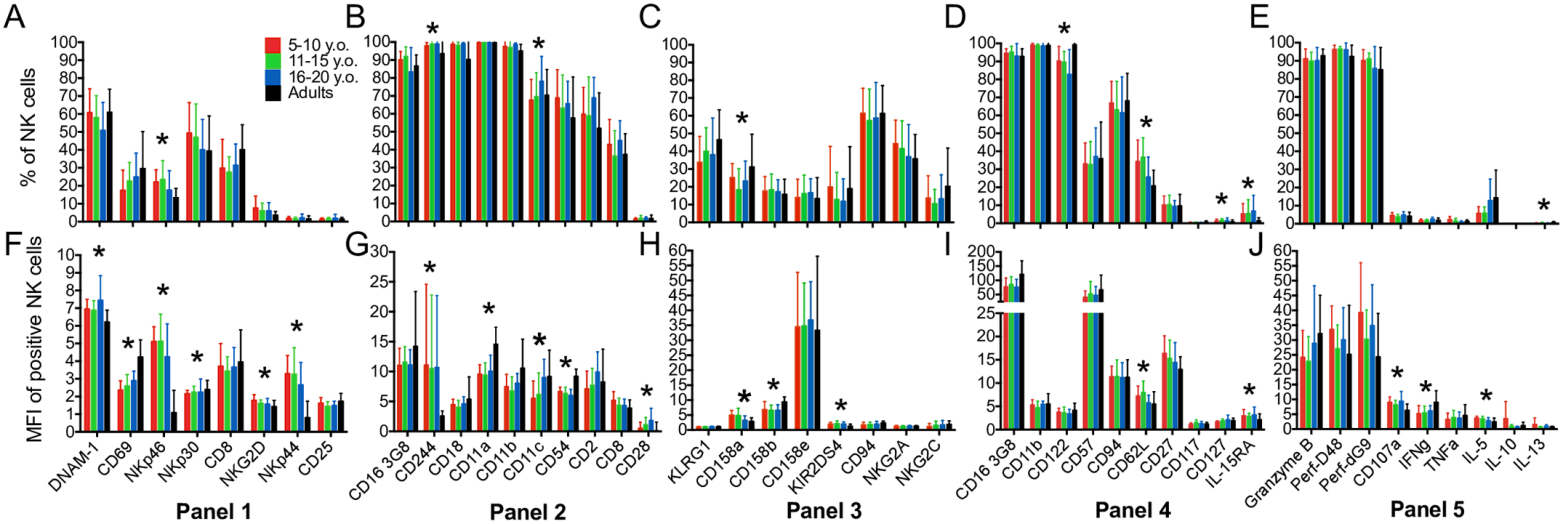
Significant inter-individual variation of NK cell markers between age groups of healthy adults and children. NK cells were defined using flow cytometry as CD3^-^CD45^high^CD56^+^ and gated above fluorescence minus one (FMO) controls for all NK cell markers (panels shown in Table 1). (A-E) Percentage of NK cells positively expressing markers without stimulation among four age groups. (F-J) Median fluorescent intensity (MFI) of NK cells with positive expression of markers without stimulation among 4 age groups. 5–10 y.o. (n = 15) (red); 11–15 y.o. (n = 15) (green); 16–20 y.o. (n = 15) (blue) and adults (n = 20) (black). All bars for Panels 1–4 are indicative of surface expression. Panel 5 markers except CD3, CD45, CD56 and CD107a were detected intracellularly as described in the Materials and methods section. All data shown is mean ± S.D. *p<0.05 among four age groups by Kruskal-Wallis and post-hoc comparison by Dunn test with Bonferroni adjustment.

Using each of these 5 panels, we compared total NK cell percentages as well as CD56^bright^ to CD56^dim^ NK cell ratios across the four age groups. In line with previous findings, the frequency of CD56^bright^ cells decreased with age while CD56^dim^ cells increased, although these trends were not significant (S2 Fig) [37, 67, 68]. Both the percentage and median fluorescent intensity (MFI) of most markers evaluated on NK cells displayed significant inter-individual variation (Fig 1) as has previously been illustrated with both flow cytometry and mass cytometry [39, 41].

Analysis of activating receptors showed that DNAM-1 was expressed on the greatest number of CD56^+^ NK cells (51–61%) while CD25 and NKp44 were the lowest (1–2%) (Fig 1A). Frequency of NKp46-positive cells was not as high as historically reported [69]. This may be due to our stringent gating strategy whereby only the brightest cells were considered positive. A recent study, however, has shown that NKp46 is found on only 25 of the top 50 NK subsets [41]. Within adhesion/ stimulatory receptors, CD16, CD11a, CD11b and CD18 were the most prevalent and expressed by 84–100% of NK cells (Fig 1B) whereas CD94 (57–61%) and NKG2A (36–44%) were highly expressed, we found that all KIR receptors had comparably lower levels of expression as would be expected (Fig 1C). It has been previously shown that NKG2C^+^ subsets expand to adult frequencies by 10 years of age, concomitant with CMV infection [70]. While we did not see any increase in percentage (data not shown), the level of NKG2C expression slightly increased in value (not significant) between 5 to 10 year-olds and 11 to 15 year-olds (S3 Fig). Mature developmental markers such as CD16, CD11b and CD122 were highly expressed by 82–99% of NK cells in all age groups as were perforin and granzyme B (85–95%). This is to be expected since total NK cells are largely representative of the CD56^dim^ subset. Conversely, but also as expected, markers highly expressed in CD56^bright^ NK cells such as CD127, CD117 and IL-15 receptor-alpha (IL-15Rα) had lower frequencies when NK cells were considered as a whole (Fig 1D). Most cytokines were also expectedly low without stimulation with the exception of IL-5 (Fig 1E).

While percentages demonstrated the frequency of positively expressing NK cells at a population level, in order to evaluate the relative intensity as a reflection of molecules per cell for markers expressed by individual NK cells, we also measured MFI (median fluorescent intensity). In general, the MFI of most markers displayed high standard deviations, unlike frequencies, where markers such as 2B4, CD18, CD11a and CD11b had very low deviations (Fig 1F to 1J). It is also important to note that MFI comparisons can only be made for the respective antibody across age groups since MFI is a property of the particular fluorochrome and antibody avidity. This is evident from the observation that the value of CD16 MFI in developmental markers panel (Fig 1I) is much higher than in adhesion/ stimulatory receptors panel (Fig 1G).

**Fig 2:**
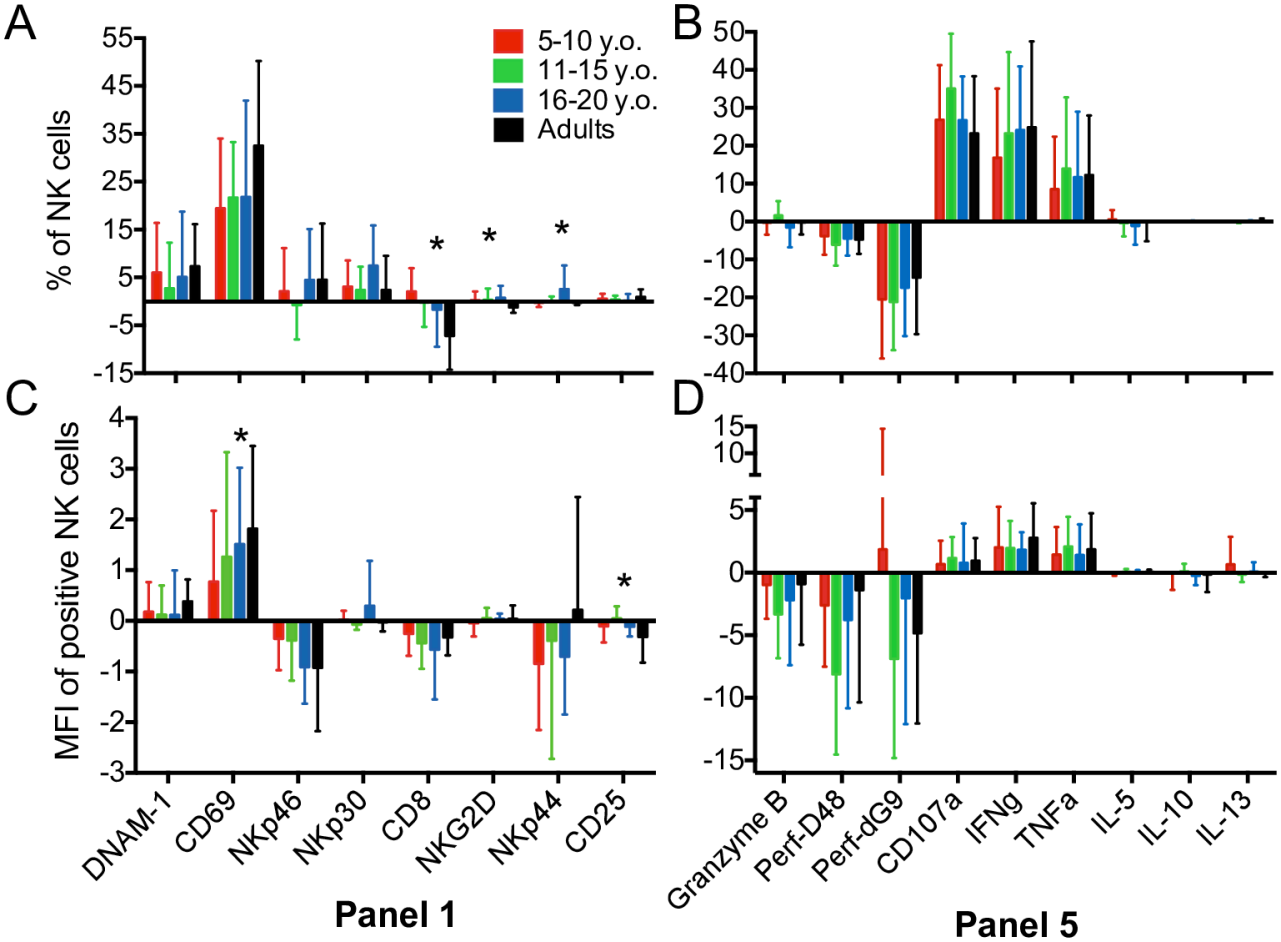
Short-term NK cell stimulation leads to significant receptor modulation. NK cells were stimulated for 4 hours using PMA/ ionomycin and evaluated using flow cytometry using panels shown in Table 1. (A-B) Percentage difference from baseline of NK cells expressing markers after stimulation among 4 age groups. (C-D) Median fluorescent intensity (MFI) difference from baseline of NK cells with positive expression of markers after stimulation among four age groups. 5–10 y.o. (n = 15) (red); 11–15 y.o. (n = 15) (green); 16–20 y.o. (n = 15) (blue) and adults (n = 20) (black). All bars for Panels 1–4 are indicative of surface expression. Panel 5 markers except CD3, CD45, CD56 and CD107a were detected intracellularly as described in the Materials and methods section. All data shown is mean ± S.D. *p<0.05 among four age groups by Kruskal-Wallis and post-hoc comparison by Dunn test with Bonferroni adjustment.

Since NK cells degranulate and upregulate cytokines and alter their expression of activation markers upon stimulation, we characterized these following stimulation with PMA and ionomycin (Panels 1 and 5). For the activating receptors panel, cells either received no stimulation or PMA/ ionomycin. However, for the cytokines and effector molecules panel, both the stimulated and unstimulated cells were treated with Brefeldin A and anti-CD107a antibody so that the spontaneous production of cytokines and degranulation of unstimulated cells could be measured. We found that the percentages of most activating receptors increased except CD8, which was slightly reduced. Interestingly, upregulation of CD69 was greatest in adults, with a seemingly progressive trend with the lowest expression in the youngest age group (5–10 y.o.) (Fig 2A). The expression of cytokines and cellular degranulation (as measured by CD107a) increased, regardless of age, while perforin and granzyme B decreased, likely due to being released on stimulation (Fig 2B). The greatest differences following stimulation were seen in CD69 (19.5–32.5%), perforin δG9 (15–21%), CD107a (23–35%), IFNγ (17–25%) and TNFα (8.5–12%), underscoring those were the most sensitive to external stimuli. In terms of MFI, a similar pattern to the percentage differences was seen. However, MFI of NKp46 and NKp44 was reduced in all groups after stimulation (Fig 2C) whereas perforin δG9 was slightly increased in only 5- to 10-year-olds (Fig 2D). Altogether, the ranges of the percent differences and MFI also had great inter-individual variation, as evident from the standard deviations. They do, however, allow considering some normative ranges. Our data expectedly revealed significant variation within several NK cell markers not only among adults but also among the heretofore unexplored pediatric population.

### Disparity between adults and children accounts for majority of the age-wise variation in NK cell markers

To better understand those markers with significant differences between the four age groups, we further examined these markers based upon their percentage expression and MFI within CD56^bright^ and CD56^dim^ NK cells. On tabulating the degree of statistical significance between the age groups, we discovered that these differences could be largely attributed to overall disparity between adults and children rather than those among the three pediatric age groups (Tables 2–5). The greatest differences within the more mature CD56^dim^ NK cells by percentage expression and MFI were found between 5- to 10-year-olds and adults (Tables 2 and 4). However, variability between markers associated with CD56^bright^ NK cells, was greatest between adolescents (11- to 15-year-olds) and adults (Tables 3 and 5) suggesting different sources of variation between the NK cell developmental stages.

**Table 2:** Significantly different markers by frequency within CD56^dim^ NK cells of healthy adults and children.

Panel	NK subset	Adults (Mean ± SD)	5–10 y.o. (Mean ± SD)	11–15 y.o. (Mean ± SD)	16–20 y.o. (Mean ± SD)	Other significant results
**Activating receptors (No stim)**	**NKp46**	10.31% ± 4.64	18.96% ± 7.06	20.39% ± 9.98	15.61% ± 10.16	
			**	***	n.s.	
**Activating receptors (With stim)**	**NKG2D**	2.06% ± 0.97	6.58% ± 5.68	5.51% ± 4.84	5.93% ± 4.76	
			*	*	**	
	**CD69**	62.38% ± 20.41	36.92% ± 18.02	44.15% ± 16.15	45.86% ± 18.82	
			**	*	n.s.	
	**DNAM-1**	67.32% ± 12.89	65.05% ± 11.79	59.46% ± 9.19	54.68% ± 17.20	
			n.s.	n.s.	*	
	**NKp44**	0.73% ± 0.54	1.36% ± 0.74	1.56% ± 0.88	4.36% ± 5.96	
			*	**	**	
**Adhesion/ stimulatory receptors**	**2B4**	94.82% ± 8.85	98.8% ± 1.01	99.39% ± 0.63	99.27% ± 0.88	
			*	***	***	
	**CD11c**	69.35% ± 14.49	65.52% ± 12.05	67.96% ± 13.63	76.80% ± 14.26	5–10 y.o. vs 16–20 y.o.
			n.s.	n.s.	n.s.	*
**Developmental markers**	**CD122**	99.50% ± 0.59	90.13% ± 8.34	89.77% ± 5.52	82.57% ± 13.85	
			***	***	***	
	**CD62L**	16.90% ± 7.32	31.95% ± 11.01	34.71% ± 10.44	23.97% ± 11.28	11–15 y.o. vs 16–20 y.o.
			***	***	n.s.	*
	**CD127**	0.65% ± 0.99	0.76% ± 0.58	1.18% ± 0.68	1.14% ± 1.36	
			n.s.	*	n.s.	
	**IL-15Rα**	1.48% ± 1.70	5.22% ± 5.49	5.42% ± 7.71	7.11% ± 9.04	
			*	n.s.	**	
**Cytokines and effector molecules (No stim)**	**IL-13**	0.85% ± 0.39	0.26% ± 0.35	0.32% ± 0.51	0.21% ± 0.22	
			***	**	***	
**Cytokines and effector molecules (With stim)**	**Perf-D48**	91.65% ± 5.43	96.05% ± 1.76	94.83% ± 3.93	95.66% ± 2.78	
			*	n.s.	*	
	**IL-13**	0.88% ± 0.68	0.28% ± 0.27	0.33% ± 0.39	0.28% ± 0.32	
			**	*	**	

Mean values represent percentage of CD56^dim^ NK cells expressing the relevant marker. Significantly different results between adults and the respective pediatric group are indicated. Other statistically significant results among 3 pediatric age groups are depicted in the last column. 5–10 y.o. (n = 15); 11–15 y.o. (n = 15); 16–20 y.o. (n = 15) and adults (n = 20).

*p<0.05 **p<0.01 and ***p<0.001 among four age groups by Kruskal-Wallis and post-hoc comparison by Dunn test with Bonferroni adjustment

**Table 3:** Significantly different markers by frequency within CD56^bright^ NK cells of healthy adults and children.

Panel	NK subset	Adults (Mean ± SD)	5–10 y.o. (Mean ± SD)	11–15 y.o. (Mean ± SD)	16–20 y.o. (Mean ± SD)	Other significant results
**Activating receptors (No stim)**	**NKp30**	53.05% ± 17.14	67.44% ± 10.9	67.15% ± 14.96	61.69% ± 14.88	
			*	*	n.s.	
	**DNAM-1**	80.17% ± 9.73	80.24% ± 11.19	76.00% ± 8.48	68.38% ± 12.11	5–10 y.o. vs 16–20 y.o.
			n.s.	n.s.	*	*
**Activating receptors (With stim)**	**NKG2D**	7.06% ± 5.86	18.68% ± 16.82	18.68% ± 11.29	18.39% ± 12.69	
			n.s.	**	**	
	**CD69**	58.12% ± 16.66	33.67% ± 23.45	44.83% ± 26.90	55.78% ± 28.65	
			**	n.s.	n.s.	
	**DNAM-1**	84.33% ± 8.33	84.17% ± 11.29	79.03% ± 9.48	71.71% ± 13.12	5–10 y.o. vs 16–20 y.o.
			n.s.	n.s.	**	**
	**NKp30**	49.53% ± 16.20	64.56% ± 10.24	61.36% ± 14.78	68.03% ± 13.78	
			*	n.s.	**	
	**NKp44**	2.91% ± 3.00	4.24% ± 3.68	3.35% ± 2.41	10.76% ± 10.70	
			n.s.	n.s.	**	
**Adhesion/ stimulatory receptors**	**2B4**	85.55% ± 13.83	90.84% ± 8.88	92.59% ± 11.05	95.52% ± 6.01	
			n.s.	n.s.	*	
	**CD11c**	84.14% ± 10.05	88.89% ± 8.40	87.3% ± 8.62	91.73% ± 6.61	
			n.s.	n.s.	**	
	**CD2**	76.97% ± 12.89	90.54% ± 8.18	83.83% ± 14.57	89.74% ± 5.61	
			**	*	n.s.	
**Developmental markers**	**CD122**	98.1% ± 1.23	95.51% ± 2.96	90.10% ± 10.10	91.32% ± 8.94	
			n.s.	*	n.s.	
	**CD117**	4.98% ± 4.89	0.96% ± 1.84	1.77% ± 2.09	3.19% ± 4.39	
			**	n.s.	n.s.	
	**CD127**	1.39% ± 1.95	8.9% ± 6.23	8.79% ± 8.03	8.82% ± 9.53	
			***	**	**	
**Cytokines and effector molecules (No stim)**	**Perf-D48**	57.71% ± 24.94	87.80% ± 10.03	83.35% ± 9.10	81.38% ± 15.05	
			***	*	*	
	**Perf-δG9**	46.19% ± 24.80	75.10% ± 12.26	68.90% ± 11.28	69.98% ± 14.33	
			***	*	*	
	**IL-5**	8.60% ± 6.89	2.34% ± 2.86	3.87% ± 4.92	6.43% ± 7.24	
			**	*	n.s.	
**Cytokines and effector molecules (With stim)**	**Perf-D48**	38.06% ± 17.34	68.92% ± 17.34	47.29% ± 22.02	52.57% ± 20.71	5–10 y.o. vs 11–15 y.o.
			***	n.s.	n.s.	*
	**Perf-δG9**	25.61% ± 14.35	58.61% ± 19.79	38.47% ± 22.95	43.67% ± 21.86	5–10 y.o. vs 11–15 y.o.
			***	n.s.	n.s.	*
	**IL-5**	6.02% ± 4.49	1.74% ± 2.35	1.56% ± 1.66	2.57% ± 3.74	
			**	**	*	

Mean values represent percentage of CD56^bright^ NK cells expressing the relevant marker. Significantly different results between adults and the respective pediatric group are indicated. Other statistically significant results among 3 pediatric age groups are depicted in the last column. 5–10 y.o. (n = 15); 11–15 y.o. (n = 15); 16–20 y.o. (n = 15) and adults (n = 20).

*p<0.05 **p<0.01 and ***p<0.001 among four age groups by Kruskal-Wallis and post-hoc comparison by Dunn test with Bonferroni adjustment.

**Table 4:** Significantly different markers by MFI within CD56^dim^ NK cells of healthy adults and children.

Panel	NK subset	Adults (Mean ± SD)	5–10 y.o. (Mean ± SD)	11–15 y.o. (Mean ± SD)	16–20 y.o. (Mean ± SD)	Other significant results
**Activating receptors (No stim)**	**NKp46**	0.83 ± 1.35	4.72 ± 0.77	4.75 ± 1.35	3.81 ± 1.77	
			***	***	***	
	**DNAM-1**	6.15 ± 0.69	6.87 ± 0.56	6.83 ± 0.54	7.4 ± 1.36	
			*	*	***	
	**CD69**	4.25 ± 0.96	2.33 ± 0.49	2.58 ± 0.64	2.89 ± 0.53	
			***	***	**	
	**NKp44**	1.50 ± 2.14	3.41 ± 1.23	3.68 ± 1.85	2.91 ± 1.23	
			***	***	**	
**Activating receptors (With stim)**	**NKp46**	0.14 ± 1.05	4.66 ± 0.96	4.60 ± 1.36	3.30 ± 1.59	
			***	***	***	
	**CD69**	6.09 ± 2.01	3.12 ± 1.80	3.84 ± 2.38	4.38 ± 1.51	
			***	**	n.s.	
	**DNAM-1**	6.55 ± 0.77	7.02 ± 0.41	6.94 ± 0.43	7.54 ± 0.66	11–15 y.o. vs 16–20 y.o.
			n.s.	n.s.	***	*
	**NKp44**	1.64 ± 1.96	2.82 ± 1.17	2.87 ± 1.65	1.93 ± 0.94	
			**	**	n.s.	
**Adhesion/ stimulatory receptors**	**2B4**	2.60 ± 0.84	11.20 ± 13.59	10.69 ± 12.58	10.94 ± 12.39	
			**	**	***	
	**CD11a**	14.66 ± 3.03	9.71 ± 1.73	9.53 ± 2.11	10.20 ± 2.80	
			***	***	***	
	**CD11b**	10.69 ± 5.12	7.53 ± 2.07	6.82 ± 2.42	8.19 ± 1.62	
			n.s.	*	n.s.	
	**CD11c**	8.36 ± 3.15	4.94 ± 2.43	5.80 ± 3.42	8.26 ± 2.67	5–10 y.o. vs 16–20 y.o.**
			**	*	n.s.	11–15 y.o. vs 16–20 y.o.*
	**CD54**	8.96 ± 1.09	6.28 ± 0.64	6.11 ± 0.96	5.81 ± 0.94	
			***	***	***	
	**CD2**	6.77 ± 4.69	5.63 ± 2.29	6.74 ± 2.96	8.58 ± 2.77	5–10 y.o. vs 16–20 y.o.
			n.s.	n.s.	n.s.	*
**Inhibitory receptors**	**CD158a**	3.11 ± 1.32	4.94 ± 1.54	4.83 ± 2.35	3.26 ± 1.44	5–10 y.o. vs 16–20 y.o.
			**	n.s.	n.s.	*
	**CD158b**	8.80 ± 2.00	6.83 ± 2.66	6.63 ± 1.72	6.61 ± 1.80	
			**	*	**	
	**KIR2DS4**	1.28 ± 0.68	2.00 ± 0.57	1.93 ± 0.87	1.99 ± 0.75	
			*	n.s.	*	
**Developmental markers**	**CD62L**	4.81 ± 2.16	6.55 ± 1.97	7.12 ± 2.01	5.57 ± 1.40	
			*	**	n.s.	
	**CD27**	11.47 ± 2.16	14.20 ± 2.94	14.30 ± 3.55	13.62 ± 3.96	
			*	*	n.s.	
	**IL-15Rα**	2.14 ± 1.20	3.04 ± 1.29	2.88 ± 0.65	3.16 ± 1.69	
			*	*	*	
**Cytokines and effector molecules (No stim)**	**IFNγ**	6.24 ± 3.07	3.50 ± 0.26	3.52 ± 0.44	4.33 ± 0.97	5–10 y.o. vs 16–20 y.o. and 11–15 y.o. vs 16–20 y.o.
			***	***	n.s.	*
	**Perf-D48**	27.31 ± 18.01	39.55 ± 7.01	37.35 ± 8.12	35.12 ± 8.66	
			*	n.s.	n.s.	
	**CD107a**	5.56 ± 1.72	9.29 ± 4.76	7.09 ± 1.72	8.97 ± 5.11	
			***	n.s.	**	
**Cytokines and effector molecules (With stim)**	**IFNγ**	9.10 ± 3.94	5.18 ± 2.54	5.51 ± 2.28	6.19 ± 1.78	
			***	**	n.s.	
	**Perf-δG9**	24.65 ± 14.67	44.11 ± 17.28	32.56 ± 10.34	37.15 ± 13.76	
			**	n.s.	n.s.	
	**CD107a**	6.39 ± 2.03	9.06 ± 1.92	8.15 ± 1.42	9.44 ± 3.20	
			**	*	**	

Mean values represent median fluorescent intensity (MFI) of CD56^dim^ NK cells positively expressing the relevant marker. Significantly different results between adults and the respective pediatric group are indicated. Other statistically significant results among 3 pediatric age groups are depicted in the last column. 5–10 y.o. (n = 15); 11–15 y.o. (n = 15); 16–20 y.o. (n = 15) and adults (n = 20).

*p<0.05 **p<0.01 and ***p<0.001 among four age groups by Kruskal-Wallis and post-hoc comparison by Dunn test with Bonferroni adjustment.

**Table 5:** Significantly different markers by MFI within CD56^bright^ NK cells of healthy adults and children.

Panel	NK subset	Adults (Mean ± SD)	5–10 y.o. (Mean ± SD)	11–15 y.o. (Mean ± SD)	16–20 y.o. (Mean ± SD)	Other significant results
**Activating receptors (No stim)**	**NKp46**	2.13 ± 2.80	7.55 ± 2.46	7.42 ± 1.69	6.71 ± 1.76	
			***	***	**	
	**CD69**	4.9 ± 1.41	3.81 ± 1.81	3.06 ± 1.1	4.07 ± 1.70	
			n.s.	**	n.s.	
**Activating receptors (With stim)**	**NKp46**	0.66 ± 2.54	5.82 ± 1.88	5.64 ± 2.00	4.16 ± 2.41	
			***	***	**	
	**CD69**	5.3 ± 1.06	3.32 ± 2.25	4.15 ± 2.54	4.82 ± 1.70	5–10 y.o. vs 16–20 y.o.
			**	n.s.	n.s.	*
	**NKp44**	5.98 ± 19.38	2.74 ± 4.39	3.68 ± 3.3	4.59 ± 6.83	
			*	**	*	
**Adhesion/ stimulatory receptors**	**2B4**	2.32 ± 0.96	9.51 ± 12.36	8.81 ± 10.58	8.01 ± 8.70	
			n.s.	*	**	
	**CD11a**	13.57 ± 2.34	8.63 ± 1.42	8.61 ± 1.95	8.38 ± 1.87	
			***	***	***	
	**CD11b**	10.41 ± 3.72	7.10 ± 2.39	6.63 ± 2.16	7.64 ± 2.42	
			*	**	n.s.	
	**CD54**	13.00 ± 2.34	11.55 ± 1.75	10.48 ± 3.23	9.32 ± 2.20	
			n.s.	*	***	
**Developmental markers**	**CD62L**	12.60 ± 7.81	21.53 ± 7.28	20.22 ± 5.62	13.88 ± 8.89	5–10 y.o. vs 16–20 y.o.
			**	*	n.s.	*
	**CD94**	35.04 ± 9.85	30.54 ± 5.95	25.95 ± 9.90	25.09 ± 8.33	
			n.s.	*	**	
**Cytokines and effector molecules (No stim)**	**IFNγ**	6.76 ± 3.45	3.67 ± 0.56	3.54 ± 0.43	4.40 ± 1.21	
			n.s.	*	n.s.	
	**CD107a**	6.13 ± 4.87	8.4 ± 3.35	11.08 ± 8.42	7.3 ± 2.50	
			*	**	n.s.	
**Cytokines and effector molecules (With stim)**	**Granzyme B**	13.48 ± 5.17	9.35 ± 6.80	9.15 ± 5.98	10.70 ± 5.93	
			*	*	n.s.	
	**IFNγ**	9.30 ± 4.19	6.06 ± 3.29	5.31 ± 2.11	6.38 ± 2.34	
			*	**	n.s.	
	**IL-5**	3.29 ± 2.55	6.41 ± 3.78	3.92 ± 1.56	3.11 ± 0.95	5–10 y.o. vs 16–20 y.o.
			*	n.s.	n.s.	*
	**CD107a**	6.32 ± 2.52	11.58 ± 8.34	9.74 ± 4.59	11.9 ± 8.41	
			***	**	**	

Mean values represent median fluorescent intensity (MFI) of CD56^bright^ NK cells positively expressing the relevant marker. Significantly different results between adults and the respective pediatric group are indicated. Other statistically significant results among 3 pediatric age groups are depicted in the last column. 5–10 y.o. (n = 15); 11–15 y.o. (n = 15); 16–20 y.o. (n = 15) and adults (n = 20).

*p<0.05 **p<0.01 and ***p<0.001 among four age groups by Kruskal-Wallis and post-hoc comparison by Dunn test with Bonferroni adjustment.

Among CD56^dim^ NK cells, the percentages of developmental markers CD122 and CD62L had the greatest variation between children of all ages and adults, while DNAM-1 showed the least significant variation (Table 2). Moreover, the frequency of CD122 expression was highest among NK cells from adults while CD62L was the least. This is consistent with our knowledge of CD56^dim^ NK cells that are CD122^high^CD62L^low^, suggesting an increase in NK cell developmental maturation that corresponds with increasing age. Interestingly, the MFI of adhesion receptors such as CD54 in CD56^dim^ NK cells (Table 4) as well as CD11a in CD56^bright^ and CD56^dim^ NK cells (Tables 4 and 5) were the most significantly different between children and adults, with the highest values in adults. Conversely, the percentage and MFI of CD11c among CD56^dim^ NK cells were the least different, but its percentage along with the MFI of CD2 in CD56^dim^ NK cells (Tables 2 and 4), exclusively differed among the pediatric groups rather than between adults and children. In addition to adhesion receptors, the MFI of NKp46 in CD56^bright^ NK cells (Table 5) and CD56^dim^ NK cells (Table 4) was the most significantly different between children and adults. Finally, upon evaluating differences in the percentages of individual CD56^bright^ subsets, perforin was the most significantly different, but only between 5- to 10-year-olds and adults (Table 3). While all panels had some markers that differed by percentage and MFI, inhibitory receptors only differed by MFI within CD56^dim^ NK cells (Table 4). In direct contrast to CD56^bright^ subsets, many more CD56^dim^ subsets significantly differed by MFI than by percentage. This suggests that the surface density of CD56^dim^ NK cell markers, unlike CD56^bright^, varies more than the overall frequency. On the whole, the significantly different markers were not consistent between percentage and MFI, thus emphasizing the importance of utilizing both measures. Differences between 16- to 20-year-olds and other pediatric groups accounted for majority of the receptors that significantly differed among children. Furthermore, the least differences in both CD56^bright^ and CD56^dim^ NK cells always existed between 16- to 20-year-olds and adults. Although the absolute values generally did not exhibit a uniform trend with age, there were more significant differences between the MFI and frequency of NK cell markers of the youngest subject group and adults, thus suggesting a progression towards adult phenotype.

**Fig 3:**
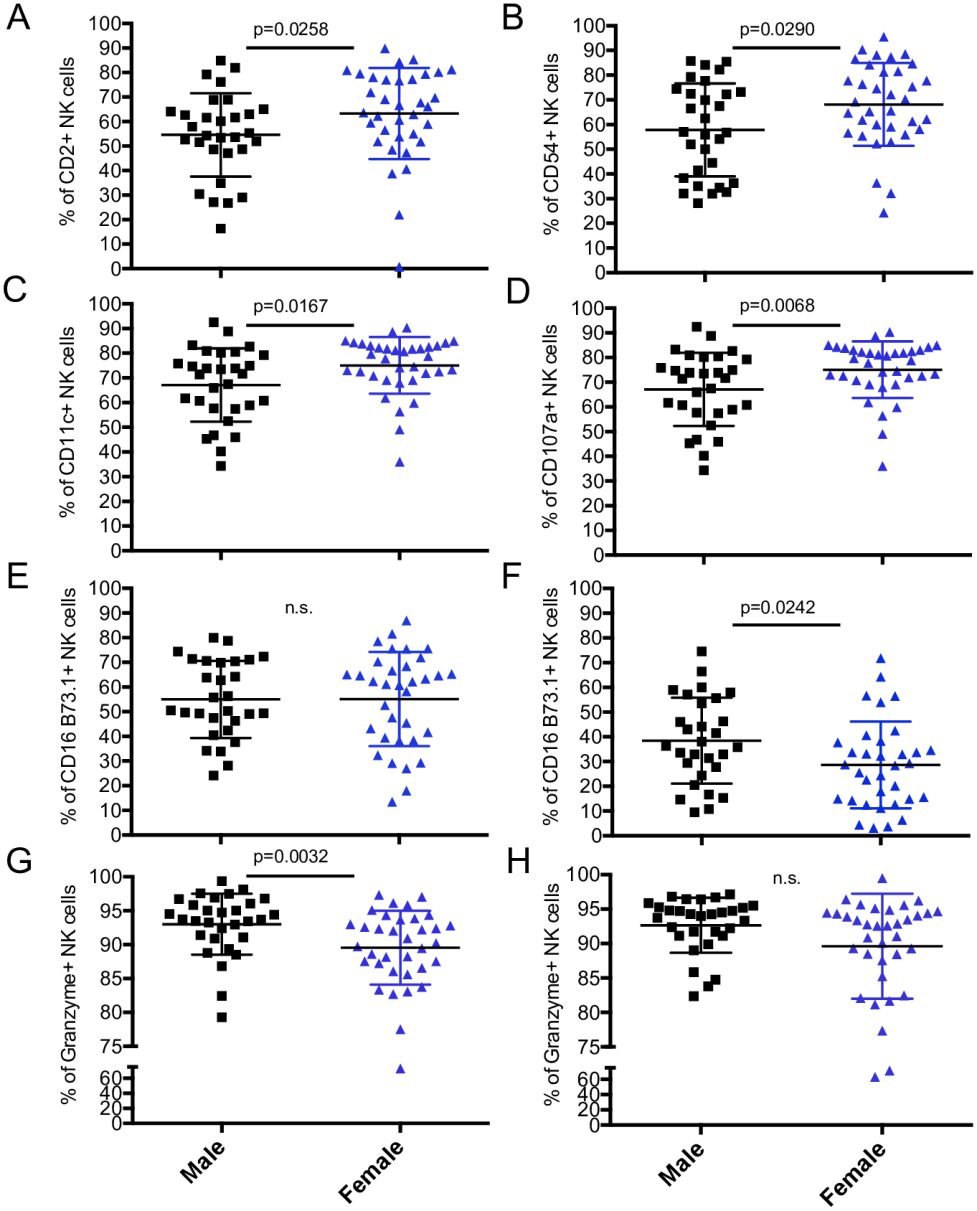
Variation in total NK cell marker expression by sex in a healthy population of adults and children. All 65 donors were pooled and divided by sex. Percentage of NK cells with positive expression of CD2, CD54 and CD11c (A-C) and CD107a after stimulation (D). CD16 (B73.1 clone) and granzyme B incubated in absence of stimulation (E, G) or stimulated with PMA/ionomycin (F, H). All markers except granzyme were detected by surface staining. Each data point represents a donor; male (n = 30), female (n = 35). All data shown is mean ± S.D. compared by Mann-Whitney test.

**Fig 4:**
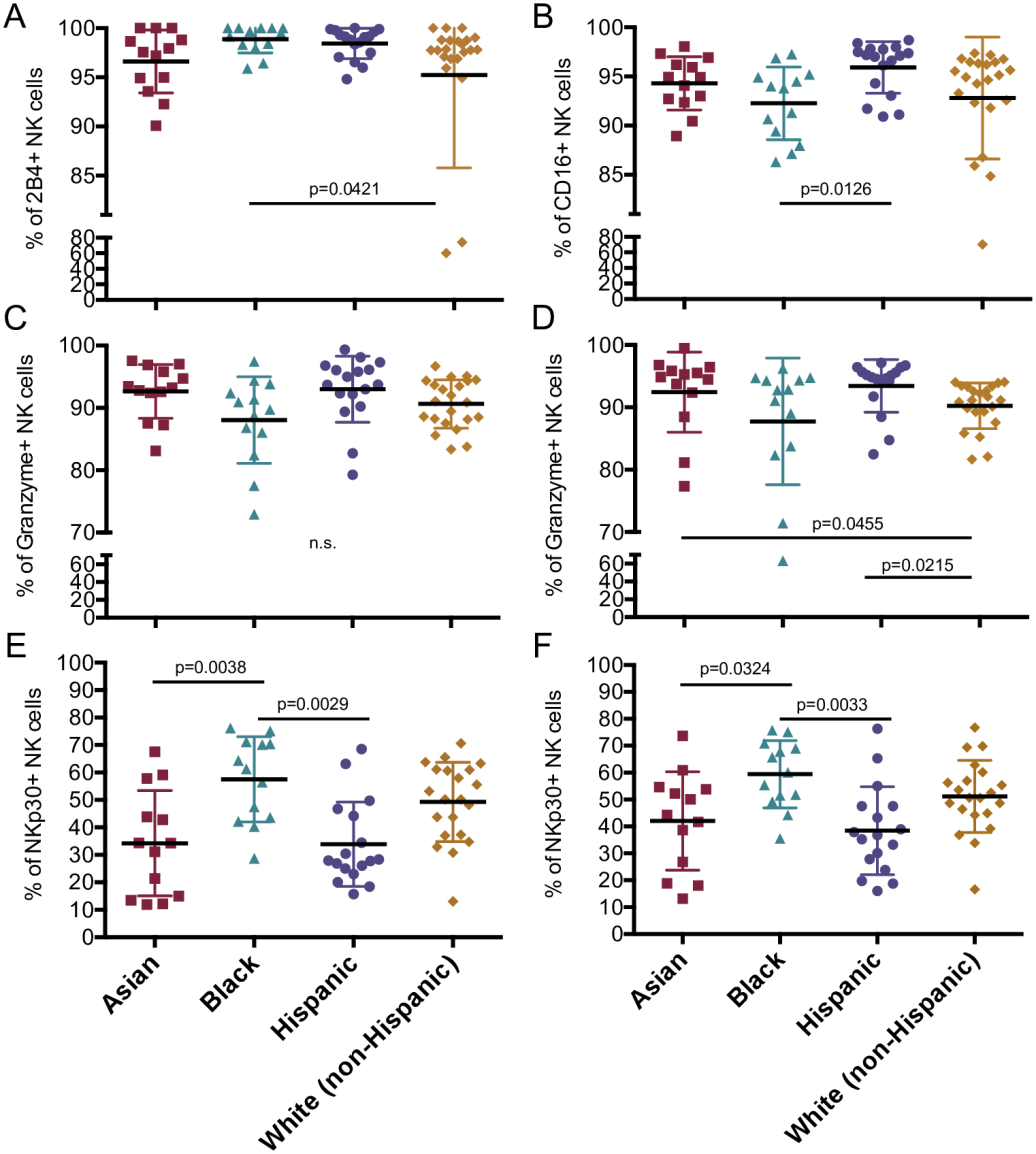
Variation in total NK cell marker expression by ethnicity in a healthy population of adults and children. All 65 donors were pooled and divided by ethnicity. Percentage of NK cells with positive expression of 2B4 and CD16 (A-B). Granzyme B and NKp30 incubated in absence of stimulation (C, E) or stimulated with PMA/ionomycin (D, F). All markers except granzyme were detected by surface staining. Each data point represents a donor; Asian (n = 13); Black (n = 13); Hispanic (n = 17) and White (non-Hispanic) (n = 21). All data shown is mean ± S.D. compared by Kruskal-Wallis and post-hoc comparison by Dunn test with Bonferroni adjustment.

As our cohort was demographically diverse, we wanted to determine if any NK cell subsets we had studied largely varied by sex or ethnicity. Although our study's small sample size limited the statistical power for these evaluations, data from adults and children were pooled and tested for significantly different subsets associated with sex or ethnicity. We found that the percentage of NK cells expressing CD16 B73.1 after stimulation and CD2, CD54, CD11c, CD107a and granzyme B without stimulation varied between the sexes (Fig 3). Except CD16 and granzyme B, females expressed most of these markers more highly than males. Granzyme B was also the most significant while CD54 was the least (Fig 3G and 3B). Other sex differences were not found to be significant. Some differences were also identified by ethnicity; specifically, the percentage of NK cells expressing 2B4, CD16, and granzyme B after stimulation and NKp30 with and without stimulation (Fig 4). Interestingly, Hispanics had either the highest or lowest expression of most of these markers except 2B4 (Fig 4A). Granzyme was the least significant while stimulated expression of NKp30 was the most (Fig 4C, 4E and 4F). While the overall distribution of study participants amongst the four ethnicities was comparable, there is a racial bias in 5- to 10-year-olds towards White (non-Hispanic). Therefore, some of the differences in NK cell receptors between 5- to 10-year-olds and adults could be due to disparity in ethnicities instead of age.

The majority (but not all) of our healthy pediatric cohort was recruited from an allergy clinic and less than half of them had food allergy. Thus, in order to determine if this might have had influence on our cell population ranges and findings, we also evaluated the frequencies of NK cell markers based on an individual’s food allergy status. The NK cell phenotype largely did not differ in children with food allergy. Among all the markers and functions evaluated, only TNFα was significantly lower in children with food allergy compared to those without food allergy and those with unknown status (S4 Fig). Overall, our results demonstrate that differences by sex, ethnicity or other identifiable cohort characteristics do not account for majority of the age-wise variation observed.

### Higher phenotypic differences in combinatorial NK subsets exist between CD56^bright^ and CD56^dim^ subsets

Thus far, sequential gating allowed us to individually determine the percentage expression of one NK cell receptor at a time. However, simultaneously examining all the receptors in each panel necessitated a distinct approach. Treeplot analysis is a data-mining tool that computes the percentages of all possible combinations of NK cell parameters per panel based on the data generated in bivariate plots. Thereby, combinatorial NK subsets that simultaneously expressed up to 8 different markers were analyzed. This was followed by the comparison of the percentages of these combinatorial subsets between adults and children (pooled) within CD56^bright^ or CD56^dim^ NK cells and between stimulated and unstimulated NK cells within adults or children. The advantage of these approaches is that they provide the opportunity to identify otherwise undetectable differences and individual subsets that can be highly characteristic of a particular cohort or stimulation. For both comparisons, there were more differences among CD56^bright^ NK cells, which is indicative of greater inherent plasticity than the more mature CD56^dim^ NK cells.

Only stimulated activating receptor subsets were found to significantly differ between adults and children (Table 6). 3 out of 4 activating receptor subsets contained DNAM-1 and CD69 and these subsets were also higher in adults. The most notable adhesion/ stimulatory receptor subset included all 8 receptors and was more highly expressed in children by both CD56^bright^ (36.97% vs. 14.95%) and CD56^dim^ (26.4% vs. 13.71%) NK cells. Developmental markers and cytokines/effector molecules only significantly varied among CD56^bright^ cells. Children possessed a greater number of CD56^bright^ NK cells that produced perforin, while adults had a higher percentage of NK cells that lacked perforin expression (Table 6).

**Table 6:** Highly significant phenotypic differences in combinatorial NK subsets between adults and children.

Cell type	Panel	NK subset	Mean Value (Adults) %	Mean Value (Children) %	% Diff
**CD56**^**bright**^	Activating receptors (With stim)	DNAM-1+CD8-CD69+NKG2D-NKp30-NKp46-NKp44-CD25-	11.2	3.9	7.3
		DNAM-1+CD8-CD69+NKG2D-NKp30-NKp46+NKp44-CD25-	4.87	1.1	3.77
**CD56**^**dim**^	Activating receptors (With stim)	DNAM-1-CD8-CD69-NKG2D-NKp30+NKp46-NKp44-CD25-	2.29	6.96	4.67
		DNAM-1+CD8+CD69+NKG2D-NKp30-NKp46-NKp44-CD25-	8.31	3.9	4.41
**CD56**^**bright**^	Adhesion/ stimulatory receptors	CD16 3G8-CD11a+CD11b+CD11c+CD18-CD54+CD244+CD2+	7.74	1.27	6.47
		CD16 3G8+CD11a+CD11b+CD11c+CD18+CD54+CD244+CD2+	14.95	36.97	22.02
**CD56**^**dim**^	Adhesion/ stimulatory receptors	CD16 3G8+CD11a+CD11b+CD11c+CD18+CD54+CD244+CD2+	13.71	26.4	12.69
**CD56**^**bright**^	Developmental markers	CD16 3G8-CD11b+CD57-CD94+CD62L-CD27-CD117-IL-15Rα-	8.25	3.55	4.7
**CD56**^**bright**^	Cytokines and effector molecules (No stim)	Perf D48-Perf δG9-Granzyme-CD107a-IFNγ-IL-5-IL-10-IL-13-	25.37	11.31	14.06
		Perf D48-Perf δG9-Granzyme+CD107a-IFNγ-IL-5-IL-10-IL-13-	9.59	1.66	7.93
		Perf D48+Perf δG9+Granzyme-CD107a-IFNγ-IL-5-IL-10-IL-13-	4.7	23.98	19.28
**CD56**^**bright**^	Cytokines and effector molecules (With stim)	Perf D48+Perf δG9+Granzyme+CD107a+IFNγ-IL-5-IL-10-IL-13-	1.71	5.74	4.03

Mean values represent percentage of CD56^bright^ or CD56^dim^ NK cells expressing the combinatorial NK subset shown. All pediatric age groups were pooled. Panels with subsets that significantly differ with p<0.0004 between adults (n = 20) and children (n = 45) are shown.

We also compared activating receptor and effector molecule subsets between stimulated and unstimulated cells within adults or children (Table 7). All CD56^bright^ activating receptor subsets included DNAM-1 and those that co-expressed CD69 were higher in stimulated cells of both adults and children. In contrast to CD56^bright^ cells, one of the most distinct subsets among CD56^dim^ cells in both adults and children were those NK cells lacking all activating receptors. This subset was found to be much higher in unstimulated cells (14.7% in children and 10.83% in adults), thus indicating that many of these receptors were induced upon activation. The majority of the CD56^bright^ cytokines and effector molecule subsets that contained perforin and granzyme B but lacked CD107a and cytokines were expectedly higher in unstimulated cells of both adults and children. Similarly, any CD56^dim^ effector subset that included CD107a or cytokines were higher in stimulated cells of children. An exception was the subset that lacked both cytokines and perforin δG9, which was more highly expressed by 13.14% of stimulated cells (Table 7). These results are consistent with the observation that while perforin and granzyme B decreased following activation (Fig 2B), a higher percentage of stimulated NK cells lack granule-associated perforin than newly synthesized perforin or granzyme.

**Table 7:** Highly significant phenotypic differences in combinatorial NK subsets between stimulated and unstimulated cells.

Cohort	Cell type	Panel	NK subset	Mean Value (With stim) %	Mean Value (No stim) %	% Diff
**Adults**	CD56^bright^	Activating receptors	DNAM-1+CD8-CD69-NKG2D-NKp30+NKp46+NKp44-CD25-	5.67	12.4	6.73
			DNAM-1+CD8-CD69+NKG2D-NKp30-NKp46-NKp44-CD25-	11.2	1.29	9.91
			DNAM-1+CD8-CD69+NKG2D-NKp30+NKp46-NKp44-CD25-	8.52	1.22	7.3
**Adults**	CD56^dim^	Activating receptors	DNAM-1-CD8-CD69-NKG2D-NKp30-NKp46-NKp44-CD25-	4.37	10.83	6.46
			DNAM-1+CD8-CD69+NKG2D-NKp30-NKp46-NKp44-CD25-	11.18	4.53	6.65
**Children**	CD56^bright^	Activating receptors	DNAM-1+CD8-CD69-NKG2D-NKp30+NKp46+NKp44-CD25-	10.03	14.11	4.08
			DNAM-1+CD8-CD69+NKG2D-NKp30+NKp46-NKp44-CD25-	6.16	1.19	4.97
			DNAM-1+CD8+CD69-NKG2D-NKp30+NKp46+NKp44-CD25-	3.65	8.33	4.68
**Children**	CD56^dim^	Activating receptors	DNAM-1-CD8-CD69-NKG2D-NKp30-NKp46-NKp44-CD25-	9.36	14.7	5.34
			DNAM-1+CD8-CD69-NKG2D-NKp30-NKp46-NKp44-CD25-	8.18	13.78	5.6
**Adults**	CD56^bright^	Cytokines and effector molecules	Perf D48+Perf δG9+Granzyme+CD107a-IFNγ-IL-5-IL-10-IL-13-	9.54	25.92	16.38
**Adults**	CD56^dim^	Cytokines and effector molecules	Perf D48+Perf δG9+Granzyme+CD107a-IFNγ-IL-5-IL-10-IL-13-	32.02	69.08	37.06
**Children**	CD56^bright^	Cytokines and effector molecules	Perf D48+Perf δG9-Granzyme-CD107a-IFNγ-IL-5-IL-10-IL-13-	4.24	10.77	6.53
			Perf D48+Perf δG9+Granzyme-CD107a-IFNγ-IL-5-IL-10-IL-13-	9.38	23.98	14.6
			Perf D48+Perf δG9+Granzyme+CD107a-IFNγ-IL-5-IL-10-IL-13-	16.96	39.22	22.26
**Children**	CD56^dim^	Cytokines and effector molecules	Perf D48+Perf δG9-Granzyme+CD107a-IFNγ-IL-5-IL-10-IL-13-	13.14	3.89	9.25
			Perf D48+Perf δG9+Granzyme+CD107a-IFNγ-IL-5-IL-10-IL-13-	33.19	77.29	44.1
			Perf D48+Perf δG9+Granzyme+CD107a-IFNγ+IL-5-IL-10-IL-13-	9.79	1.32	8.47
			Perf D48+Perf δG9+Granzyme+CD107a+IFNγ-IL-5-IL-10-IL-13-	11.92	2.12	9.8
			Perf D48+Perf δG9+Granzyme+CD107a+IFNγ+IL-5-IL-10-IL-13-	9.03	0.08	8.95

Mean values represent percentage of CD56^bright^ or CD56^dim^ NK cells expressing the combinatorial NK subset shown. All pediatric groups were pooled and all comparisons were made within adults (n = 20) or within children (n = 45). Panels with subsets that significantly differed at p<0.0001 between stimulated and unstimulated cells are shown.

Finally, we distinguished CD56^bright^ and CD56^dim^ subsets across all panels (Table 8). The aforementioned subset that lacked all activating receptors was higher in CD56^dim^ NK cells in both adults and children. Conversely, all subsets that were expressed more highly by CD56^bright^ NK cells included DNAM-1, NKp30 and NKp46. The subset that contained all the adhesion/ stimulatory receptors except CD16 was higher among CD56^bright^ NK cells whereas all subsets that included CD16, CD11a, CD11b, CD18 and 2B4 were higher in CD56^dim^ cells. However, the subset that contained all the adhesion/ stimulatory receptors was larger within the CD56^bright^ (37.0%) as opposed to CD56^dim^ population (26.4%). Inhibitory receptor subsets that differed between CD56^bright^ and CD56^dim^ NK cells lacked all CD158 receptors as might have been predicted owing to the known association between KIR expression and terminal maturation. One of the highest frequency inhibitory receptor subsets that was significantly higher in CD56^bright^ NK cells (33.3% in adults and 34.8% in children) only included CD94 and NKG2A. Contrarily, subsets that were higher in CD56^dim^ NK cells also contained KIR2DS4 or NKG2C. All developmental marker panel subsets that were higher in CD56^bright^ NK cells included CD11b, CD94 and CD62L but lacked CD57, CD117 and IL-15Rα. On the other hand, all subsets that were higher in CD56^dim^ NK cells contained CD16 and CD11b while some also co-expressed CD57. These results are also generally consistent with our knowledge of NK cell biology, as CD56^dim^ NK cells are more mature and CD57-positive cells are considered terminally differentiated [2]. All cytokine and effector molecule subsets that were higher in CD56^dim^ NK cells contained perforin and granzyme in direct contrast with CD56^bright^ NK cells. Intriguingly, the subset that lacked all cytokines and effector molecules was also higher in CD56^bright^ NK cells in both adults and children. Altogether, developmental marker subsets represented the most differences between CD56^bright^ and CD56^dim^ NK cells. Moreover, majority of the significant phenotypic differences in combinatorial NK subsets occurred when contrasting CD56^bright^ and CD56^dim^ subsets within cohorts than between adults and children or stimulated and unstimulated cells (Table 8). Thus, our collective analysis findings clearly emphasize the distinct developmental stages that these two subsets represent. As such, more differences exist between them than adults and children on the whole.

**Table 8:** Highly significant phenotypic differences in combinatorial NK subsets between CD56^bright^ and CD56^dim^ cells.

Cohort	Panel	NK subset	Mean Value (CD56^bright^) %	Mean Value (CD56^dim^) %	% Diff
**Adults**	Activating receptors (No stim)	DNAM-1-CD8-CD69-NKG2D-NKp30-NKp46-NKp44-CD25-	1.94	10.83	8.89
		DNAM-1+CD8-CD69-NKG2D-NKp30-NKp46-NKp44-CD25-	7.68	14.08	6.4
		DNAM-1+CD8-CD69-NKG2D-NKp30+NKp46+NKp44-CD25-	12.4	1.72	10.68
**Children**	Activating receptors (No stim)	DNAM-1-CD8-CD69-NKG2D-NKp30-NKp46-NKp44-CD25-	3.67	14.7	11.03
		DNAM-1-CD8-CD69-NKG2D-NKp30+NKp46-NKp44-CD25-	3.33	8.29	4.96
		DNAM-1+CD8-CD69-NKG2D-NKp30-NKp46-NKp44-CD25-	5.48	13.78	8.3
		DNAM-1+CD8-CD69-NKG2D-NKp30+NKp46+NKp44-CD25-	14.11	4.34	9.77
		DNAM-1+CD8-CD69-NKG2D+NKp30+NKp46+NKp44-CD25-	4.79	0.27	4.52
		DNAM-1+CD8+CD69-NKG2D-NKp30+NKp46+NKp44-CD25-	8.33	1.43	6.9
**Children**	Activating receptors (With stim)	DNAM-1-CD8-CD69-NKG2D-NKp30-NKp46-NKp44-CD25-	2.86	9.36	6.5
		DNAM-1-CD8-CD69-NKG2D-NKp30+NKp46-NKp44-CD25-	3.02	6.96	3.94
		DNAM-1+CD8-CD69-NKG2D-NKp30+NKp46+NKp44-CD25-	10.03	4.84	5.19
**Adults**	Adhesion/ stimulatory receptors	CD16 3G8-CD11a+CD11b+CD11c+CD18+CD54+CD244+CD2+	18.29	1.76	16.53
		CD16 3G8+CD11a+CD11b+CD11c+CD18+CD54-CD244+CD2-	3.74	14.43	10.69
**Children**	Adhesion/ stimulatory receptors	CD16 3G8-CD11a+CD11b+CD11c+CD18+CD54+CD244+CD2+	28.57	1.8	26.77
		CD16 3G8+CD11a+CD11b+CD11c-CD18+CD54+CD244+CD2+	3.95	11.28	7.33
		CD16 3G8+CD11a+CD11b+CD11c+CD18+CD54-CD244+CD2-	1.25	10.36	9.11
		CD16 3G8+CD11a+CD11b+CD11c+CD18+CD54-CD244+CD2+	2.38	12.64	10.26
		CD16 3G8+CD11a+CD11b+CD11c+CD18+CD54+CD244+CD2-	4.81	14.17	9.36
		CD16 3G8+CD11a+CD11b+CD11c+CD18+CD54+CD244+CD2+	36.97	26.4	10.57
**Adults**	Inhibitory receptors	CD158a-CD158b-CD158e-KIR2DS4-CD94+NKG2A+NKG2C-KLRG1-	33.3	8.35	24.95
**Children**	Inhibitory receptors	CD158a-CD158b-CD158e-KIR2DS4-CD94-NKG2A-NKG2C-KLRG1-	2.28	7.55	5.27
		CD158a-CD158b-CD158e-KIR2DS4-CD94-NKG2A-NKG2C-KLRG1+	1.19	6.5	5.31
		CD158a-CD158b-CD158e-KIR2DS4-CD94+NKG2A+NKG2C-KLRG1-	34.83	12.08	22.75
		CD158a-CD158b-CD158e-KIR2DS4-CD94+NKG2A+NKG2C-KLRG1+	6.27	11.87	5.6
		CD158a-CD158b-CD158e-KIR2DS4-CD94+NKG2A+NKG2C+KLRG1-	5.57	0.4	5.17
		CD158a-CD158b-CD158e-KIR2DS4+CD94+NKG2A+NKG2C-KLRG1-	6.67	1.42	5.25
**Adults**	Developmental markers	CD16 3G8-CD11b+CD57-CD94+CD62L+CD27-CD117-IL-15Rα-	15.36	0.28	15.08
		CD16 3G8+CD11b+CD57-CD94-CD62L-CD27-CD117-IL-15Rα-	1.38	14.64	13.26
		CD16 3G8+CD11b+CD57-CD94+CD62L-CD27-CD117-IL-15Rα-	8.76	27.99	19.23
		CD16 3G8+CD11b+CD57-CD94+CD62L+CD27+CD117-IL-15Rα-	16.54	1.66	14.88
		CD16 3G8+CD11b+CD57+CD94+CD62L-CD27-CD117-IL-15Rα-	3.47	19.89	16.42
**Children**	Developmental markers	CD16 3G8-CD11b+CD57-CD94+CD62L+CD27-CD117-IL-15Rα-	7.52	0.23	7.29
		CD16 3G8-CD11b+CD57-CD94+CD62L+CD27+CD117-IL-15Rα-	9.41	0.22	9.19
		CD16 3G8+CD11b+CD57-CD94-CD62L-CD27-CD117-IL-15Rα-	2.4	14.33	11.93
		CD16 3G8+CD11b+CD57-CD94+CD62L-CD27-CD117-IL-15Rα-	6.17	19.81	13.64
		CD16 3G8+CD11b+CD57-CD94+CD62L+CD27-CD117-IL-15Rα-	19	12.8	6.2
		CD16 3G8+CD11b+CD57-CD94+CD62L+CD27+CD117-IL-15Rα-	19.18	2.38	16.8
		CD16 3G8+CD11b+CD57+CD94-CD62L-CD27-CD117-IL-15Rα-	2.28	10.33	8.05
		CD16 3G8+CD11b+CD57+CD94+CD62L-CD27-CD117-IL-15Rα-	5.36	14.32	8.96
**Adults**	Cytokines and effector molecules (No stim)	Perf D48-Perf δG9-Granzyme-CD107a-IFNγ-IL-5-IL-10-IL-13-	25.37	1.02	24.35
		Perf D48+Perf δG9+Granzyme+CD107a-IFNγ-IL-5-IL-10-IL-13-	25.92	69.08	43.16
**Children**	Cytokines and effector molecules (No stim)	Perf D48-Perf δG9-Granzyme-CD107a-IFNγ-IL-5-IL-10-IL-13-	11.31	1.3	10.01
		Perf D48+Perf δG9-Granzyme-CD107a-IFNγ-IL-5-IL-10-IL-13-	10.77	1.08	9.69
		Perf D48+Perf δG9+Granzyme-CD107a-IFNγ-IL-5-IL-10-IL-13-	23.98	2.07	21.91
		Perf D48+Perf δG9+Granzyme+CD107a-IFNγ-IL-5-IL-10-IL-13-	39.22	77.29	38.07
**Adults**	Cytokines and effector molecules (With stim)	Perf D48-Perf δG9-Granzyme-CD107a-IFNγ-IL-5-IL-10-IL-13-	20.06	1.05	19.01
		Perf D48-Perf δG9-Granzyme+CD107a-IFNγ-IL-5-IL-10-IL-13-	11.16	0.81	10.35
		Perf D48+Perf δG9+Granzyme+CD107a-IFNγ-IL-5-IL-10-IL-13-	9.54	32.02	22.48
		Perf D48+Perf δG9+Granzyme+CD107a-IFNγ+IL-5-IL-10-IL-13-	2.03	12.14	10.11
**Children**	Cytokines and effector molecules (With stim)	Perf D48-Perf δG9-Granzyme-CD107a-IFNγ-IL-5-IL-10-IL-13-	12.08	1.24	10.84
		Perf D48-Perf δG9-Granzyme+CD107a-IFNγ-IL-5-IL-10-IL-13-	6.35	0.33	6.02
		Perf D48+Perf δG9-Granzyme+CD107a-IFNγ-IL-5-IL-10-IL-13-	3.51	13.14	9.63
		Perf D48+Perf δG9+Granzyme-CD107a-IFNγ-IL-5-IL-10-IL-13-	9.38	1.1	8.28
		Perf D48+Perf δG9+Granzyme+CD107a-IFNγ-IL-5-IL-10-IL-13-	16.96	33.19	16.23
		Perf D48+Perf δG9+Granzyme+CD107a-IFNγ+IL-5-IL-10-IL-13-	3.06	9.79	6.73
		Perf D48+Perf δG9+Granzyme+CD107a+IFNγ-IL-5-IL-10-IL-13-	5.74	11.92	6.18
		Perf D48+Perf δG9+Granzyme+CD107a+IFNγ+IL-5-IL-10-IL-13-	3.32	9.03	5.71

Mean values represent percentage of CD56^bright^ or CD56^dim^ NK cells expressing the combinatorial NK subset shown. All pediatric groups were pooled and all comparisons were made within adults (n = 20) or within children (n = 45). Panels with subsets that significantly differed at p<0.0001 between CD56^bright^ and CD56^dim^ NK cells are shown.

Overall, among differences in frequency of unstimulated NK cells of children compared to adults (Table 9, S3 Table), the combinatorial subset containing 8 adhesion/ stimulatory receptors was more prevalent in children (Table 9A). Both NKp46 and CD62L in CD56^dim^ NK cells as well as perforin-containing subsets in CD56^bright^ NK cells are also higher in children. MFI of NKp46 and CD62L was increased in adults, similar to their frequencies (Table 9B). Other notable markers that were significantly higher in children include 2B4 and CD107a. However, MFI of CD69, CD11c and IFNγ were much lower in children.

**Table 9:** Most relevant markers and combinatorial subsets in unstimulated NK cells.

Value	NK cell type	Distinguishing subset/ marker	5–10 y.o.	11–15 y.o.	16–20 y.o.
**A**	**Percent change in NK cell frequency of pediatric cohort compared to adults**				
**%**	Total	**CD16 3G8+CD11a+CD11b+CD11c+CD18+CD54+CD244+CD2+**	↑		
	CD56^dim^	NKp46		↑	
		CD62L	↑	↑	
	CD56^bright^	Perforin	↑↑↑		
		Perf D48-Perf δG9-Granzyme-CD107a-IFNγ-IL-5-IL-10-IL-13-	↓		
		**Perf D48+Perf δG9+**Granzyme-CD107a-IFNγ-IL-5-IL-10-IL-13-	↑↑		
**B**	**Fold change in NK cell MFI of pediatric cohort compared to adults**				
**MFI**	Total	NKp46	↑↑	↑↑	↑↑
	CD56^dim^	CD69	↓		
		2B4	↑↑	↑↑	↑↑
		CD11c	↓		
		CD158a	↑		
		CD62L		↑	
		CD107a	↑		↑
		IFNγ	↓	↓	
	CD56^bright^	2B4			↑↑
		CD107a		↑	

(A) Arrows represent percent change in NK cells expressing the marker or combinatorial subset shown with adults as baseline. Each marker/ subset has a percent change of >10%, fold change >0.4 and p<0.01 from adults. Those age groups that did not fulfill each of these criteria have been left blank. One arrow = percent change of 10–19%; two arrows = percent change of 19–28%; three arrows = percent change of 28–37%. (B) Arrows represent fold change in NK cells expressing the marker shown with adults as baseline. Each marker has a fold change >0.4 and p<0.01 from adults. Those age groups that did not fulfill each of these criteria have been left blank. One arrow = fold change of 0.4–2; two arrows = fold change of 2–5.

We also tabulated the most relevant modulation of NK cell markers after stimulation (Table 10, S4 Table). The increase in CD69 following stimulation was at least 10% more in adults than in the youngest children (Table 10A). However, fold changes in MFI were more similar between adults and children (Table 10B). Although MFI values of stimulated cells significantly differed in adults and children, the comparable fold changes highlight the disparate values of unstimulated cells (Tables 4 and 5). Thus, majority of the most relevant differences between children and adults are at baseline without stimulation. Moreover, given the aforementioned distinction between CD56^bright^ and CD56^dim^ NK cells (Table 8), the markers were mostly non-overlapping between these developmental subsets.

**Table 10:** Most relevant markers and combinatorial subsets in stimulated NK cells.

Value	NK cell type	Distinguishing marker	5–10 y.o.	11–15 y.o.	16–20 y.o.	Adults
**A**	**Percent change in NK cell frequency of adults and children after stimulation**					
**%**	CD56^dim^	CD69	↑↑			↑↑↑
	CD56^bright^	CD69	↑↑			↑↑↑↑
**B**	**Fold change in NK cell MFI of adults and children after stimulation**					
**MFI**	Total	NKp46	<0.4↓	<0.4↓	<0.4↓	↓
	CD56^dim^	CD69	<0.4↑	↑		↑
		IFNγ	↑	↑		↑
	CD56^bright^	CD107a	<0.4↑	<0.4↓	↑	<0.4↑
		IFNγ		↑		<0.4↑

(A) Arrows represent percent change in NK cells expressing the marker shown with unstimulated cells of the respective age group as baseline. Each marker has a percent change of >10% and fold change >0.4 from unstimulated cells. Only those markers that significantly differed at p<0.01 between stimulated NK cells of adults and the respective pediatric groups were considered. Those age groups that did not fulfill each of the above criteria have been left blank. One arrow = percent change of 10–19%; two arrows = percent change of 19–28%; three arrows = percent change of 28–37%; four arrows = percent change of 37–46%. (B) Arrows represent fold change in NK cells expressing the marker shown with unstimulated cells of the respective age group as baseline. Only those markers that significantly differed at p<0.01 between stimulated NK cells of adults and the respective pediatric groups were considered. Those age groups that did not significantly differ have been left blank. Markers with a fold change <0.4 from unstimulated cells but p<0.01 from adults are indicated accordingly. One arrow = fold change of 0.4–2.

### Clustering analysis and overall diversity reveal differences in all age groups

**Fig 5:**
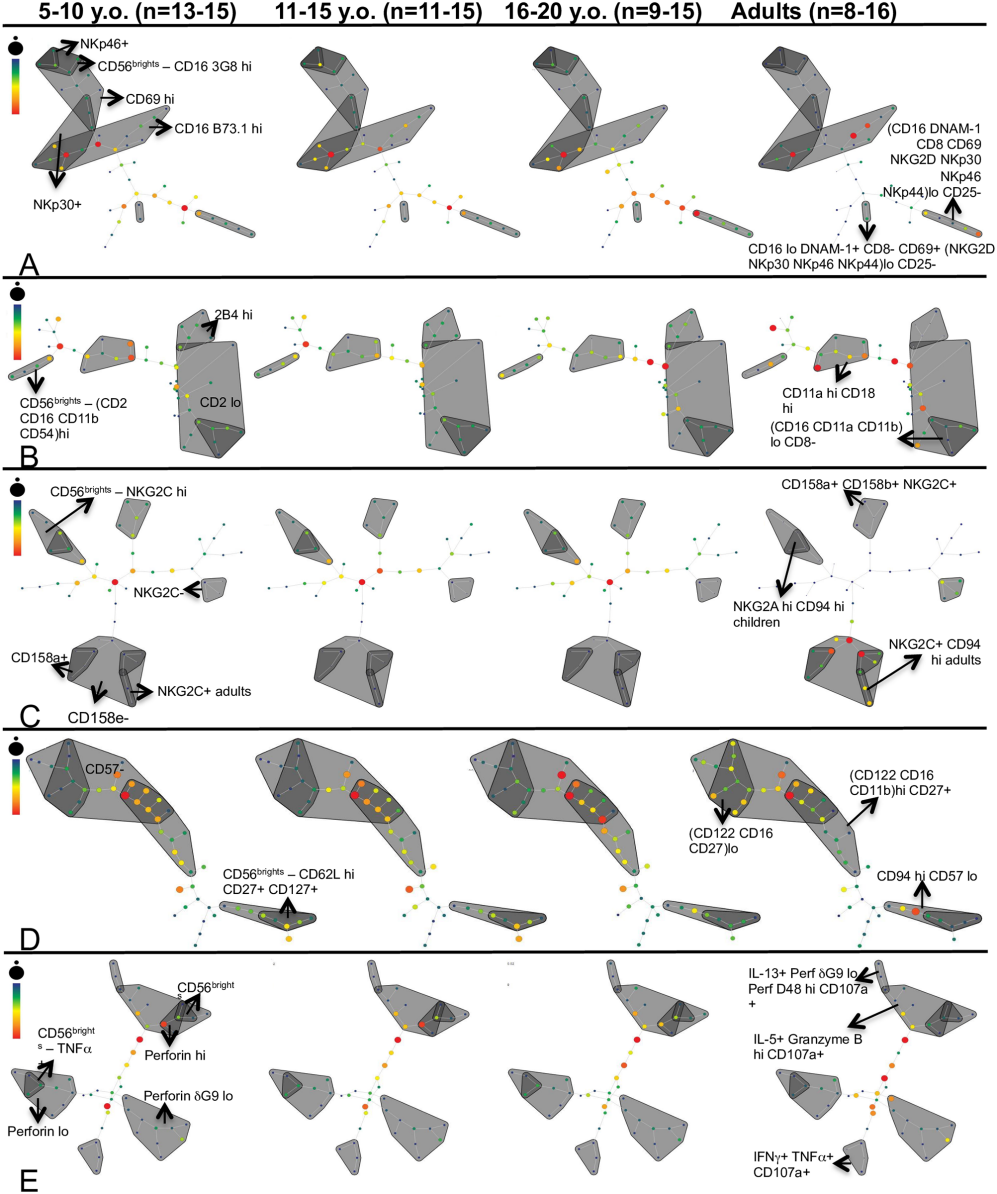
Clustering analysis of NK cell subsets using SPADE demonstrates phenotypic differences in healthy adults and children. Each SPADE plot illustrates the concatenated NK cell data from several individuals and each node corresponds to the percentage of NK cells. Color of each node represents signal intensity (see scale) and size represents frequency. The regions of the plot (grey) were characterized by sequentially defining expression of each panel marker by signal intensity in separate SPADE plots. Individual markers defining entire regions are shown in the leftmost plot and combinatorial NK subset regions are indicated in the rightmost plot. (A) NK cells expressing activating receptors without stimulation among 5–10 y.o. (n = 15), 11–15 y.o. (n = 15), 16–20 y.o. (n = 15) and adults (n = 8) respectively. (B) NK cells expressing adhesion/ stimulatory receptors among 5–10 y.o. (n = 14), 11–15 y.o. (n = 14), 16–20 y.o. (n = 15) and adults (n = 12) respectively. (C) NK cells expressing inhibitory receptors among 5–10 y.o. (n = 15), 11–15 y.o. (n = 15), 16–20 y.o. (n = 15) and adults (n = 16) respectively. (D) NK cells expressing developmental markers among 5–10 y.o. (n = 13), 11–15 y.o. (n = 11), 16–20 y.o. (n = 9) and adults (n = 12) respectively. (E) NK cells expressing cytokines and effector molecules without stimulation among 5–10 y.o. (n = 15), 11–15 y.o. (n = 15), 16–20 y.o. (n = 15) and adults (n = 8) respectively.

Using treeplot analysis, we were able to quantify the distribution of several combinatorial subsets among CD56^bright^ and CD56^dim^ NK cells. However, treeplot analysis relies on manually performed hierarchical gating, which may introduce a degree of bias. Consequently, another approach was employed to define combinatorial subsets in a more unsupervised manner with the goal of further identifying any otherwise hidden subsets characteristic of a particular cohort or stimulation. Spanning-tree progression analysis of density-normalized events (SPADE) converts high-dimensional multivariate data into two-dimensional data and clusters phenotypically similar cells together in nodes [46]. Additionally, neighboring nodes are more likely to share similar properties than distant ones, which enables definition of regions of the SPADE plot. Each node has two attributes—color and size. Color represents signal intensity, represented by the scale, and size represents frequency [46]. Using SPADE, we created panel-specific plots for each of our four age groups. Every SPADE plot illustrates the concatenated NK cell data from several individuals and each node corresponds to the percentage of NK cells (Fig 5). Distinguishing the regions in the SPADE plot allowed us to detect trends across the age groups. For instance, the percentage of NK cells that had low expression of all activating receptors except CD25, which was absent, steadily increased from 5- to 10-year-olds to adults (Fig 5A). This was evident from the greater number of nodes in this region that were brighter in adults. Among adhesion/stimulatory receptor plots, the percentage of NK cells with low expression of CD2 also increased from childhood to adulthood (Fig 5B). This is in concert with our previous results where we showed that the MFI of CD2 declined in adults (Fig 1G). The alterations in the overlapping region can also be compared along with the CD2-low region. A higher percentage of NK cells in adults lacked CD158e in inhibitory receptor plots. The percentage of NK cells that expressed CD158a, within the CD158e-negative region, also increased in adults (Fig 5C). Among developmental marker plots, the percentage of NK cells with low expression of CD122, CD16 and CD27 was greater in adults (Fig 5D). Finally, when contrasting plots generated based on cytokine and effector molecules, a higher percentage of NK cells in adults had low expression of perforin δG9 as opposed to the subsets expressing IL-5, granzyme B and CD107a, which decreased (Fig 5E). This would imply that these NK cells would have the least cytotoxic potential in adults.

While there were phenotypic differences between all age groups, majority nodes only exhibited subtle changes in color across the groups, which is unsurprising given that these are all healthy individuals. However, the plots for all panels, except adhesion/ stimulatory receptors, displayed greatest phenotypic similarity among the three pediatric age groups, reinforcing the results from other analyses (Tables 2–5). Furthermore, SPADE analysis also identified previously undetected combinatorial subsets that varied in their intensity of expression of the NK cell markers, such as the perforin-low regions (Fig 5E), thereby highlighting the usefulness of SPADE. In this manner, we qualitatively compared the distinct regions of the SPADE plots and identified emerging patterns, which revealed differences in both rare and abundant NK cell subsets.

**Fig 6:**
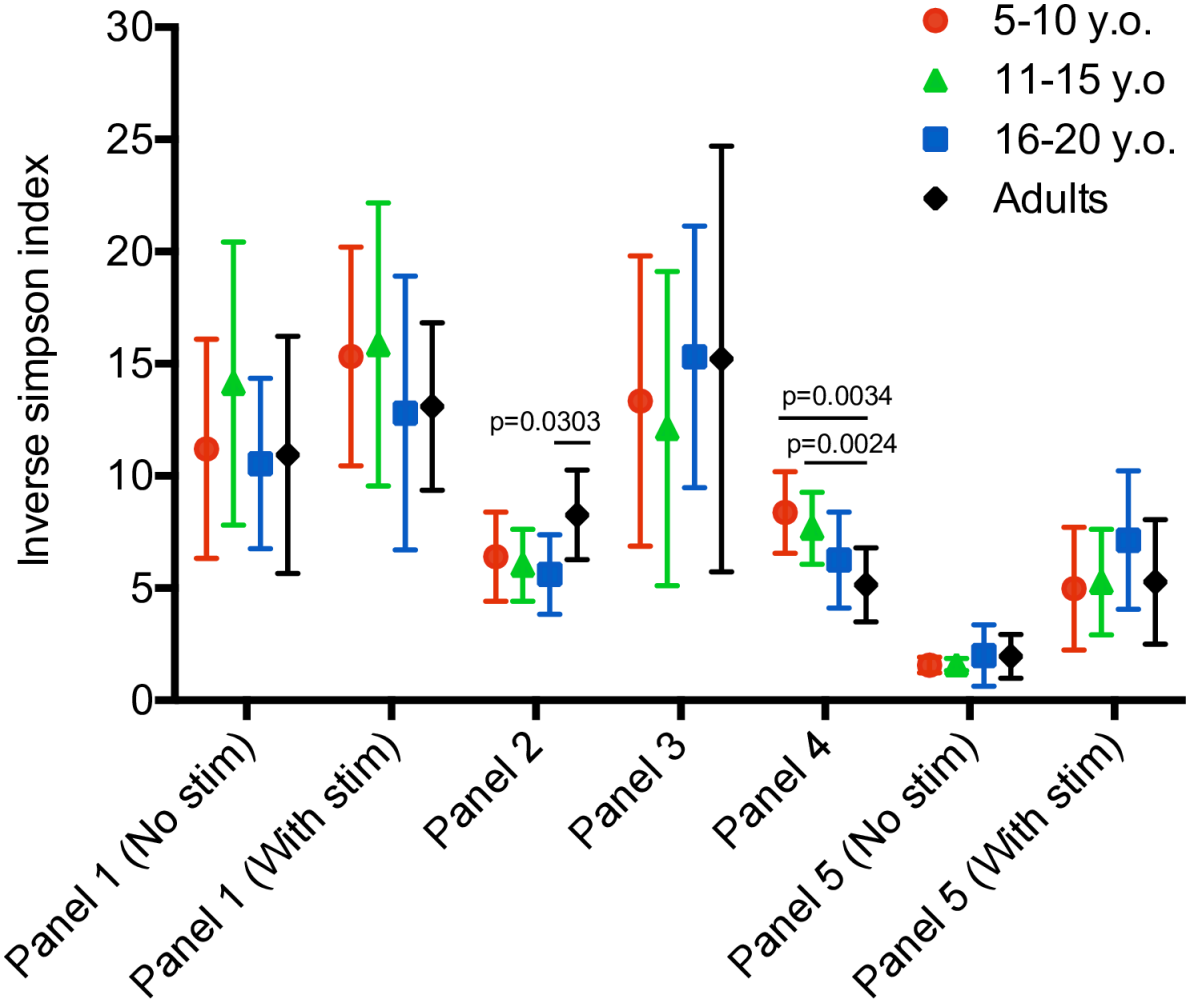
Quantification of NK cell diversity based on combinatorial expression of individual receptors. Inverse Simpson indices are indicated for each panel and stratified by age group. Index values are representative of total number of distinct panel-wise combinatorial NK subsets found in each donor (diversity). The combinatorial subsets were previously computed using treeplot analysis. All data shown is median ± S.D. 5–10 y.o. (n = 15) (red); 11–15 y.o. (n = 15) (green); 16–20 y.o. (n = 15) (blue) and adults (n = 20) (black).

Having quantified the top most significant combinatorial subsets using treeplot analysis, we next sought to quantify the level of diversity by age group for every panel. In order to do this, we calculated the inverse Simpson diversity index, which has previously been used as a measure of NK cell diversity [41]. This index takes into account both the distribution of the species and their total abundance within a population. For the purpose of this analysis, each combinatorial phenotype represented a species, that constituted organisms represented by individual NK cells [41]. Thus, the collection of subsets that belonged to a donor made up its phenotypic diversity. Using this index, we discovered that diversity between age groups is similar for Panels 1, 3 and 5, although, adults had the highest adhesion/stimulatory receptor (Panel 2) diversity and the least developmental marker (Panel 4) diversity. Interestingly, developmental marker diversity progressively diminished from 5 to 10 year-olds to adults. Also, overall diversity values were lowest for cytokines and effector molecules (Panel 5) and highest for inhibitory receptors (Panel 3) (Fig 6). This is not unexpected given that perforins and granzyme generally coexist within cells. Furthermore, the inhibitory receptor panel mostly comprises KIRs, whose extensive polymorphism is well established [71]. Since KIR repertoire can greatly vary between individuals, it naturally follows that Panel 3 should have the highest diversity among our panels. Thus, not only were we able to evaluate the percentages of several NK combinatorial subsets, but we also determined the NK cell repertoire diversity and its distinctions by age which in most but not all cases diminishes.

## Discussion

NK cell phenotype is governed by genetic and environmental components, leading to demonstrated heterogeneity within healthy adults. Despite the effect of NK cell phenotype on function, the phenotypic diversity within young children and adolescents has not been examined. We sought to define NK cell phenotype in healthy children and adults using multi-parametric flow cytometry with a goal of defining both trends and some normal ranges. Here, we established key differences between adults and children in receptor surface density and frequency, combinatorial subset expression, and diversity indices. To our knowledge, this is the first extended phenotyping of NK cells from healthy children. Given the known effect of primary immunodeficiency on NK cell subsets and frequencies [72], understanding the range and variability will be key to contextualizing aberrant NK cell phenotype in several diseases.

We phenotyped total NK cells as well as the CD56^bright^ and CD56^dim^ NK cell subsets. We found that, consistent with previous reports, the frequency of CD56^bright^ NK cells steadily declines with age while that of CD56^dim^ NK cells reciprocally rises [37, 67, 68]. The relative ratios of CD56^bright^ and CD56^dim^ NK cells have consequences in various diseases. Increased proportion of CD56^bright^ NK cells has been reported in individuals with recalcitrant warts, hepatitis C virus, tuberculin skin test positivity, systemic lupus erythematosus [73–76] and is also associated with positive outcomes in the treatment of multiple sclerosis [77]. Conversely, reduced CD56^bright^ NK cell frequencies are seen in coronary heart disease, allergic rhinitis/ asthma and juvenile rheumatoid arthritis [78–80]. Our data also highlights the differences between CD56^bright^ and CD56^dim^ NK cells. There were twice as many CD56^bright^ and CD56^dim^ NK cell combinatorial subsets (Table 8) that were significantly different than between adults and children or stimulated and unstimulated NK cells (Tables 6 and 7). Since CD56^bright^ and CD56^dim^ NK cells are discrete developmental subsets, developmental markers panel expectedly had the greatest number of significantly different combinatorial subsets. These results further underscore the importance of examining both CD56^bright^ and CD56^dim^ NK cells.

Analyzing the combinatorial NK cell subsets also allowed us to identify intermediate subsets between CD56^bright^ and CD56^dim^ NK cells. Although CD56^bright^ NK cells are primarily considered cytokine-producing and not cytotoxic, a previous study has found CD56^bright^ CD16+ NK cells that are phenotypic and functional intermediaries [31]. Indeed, our treeplot analysis revealed CD56^bright^ subsets that generated perforin and granzyme B, albeit fewer than CD56^dim^ NK cells. Conversely, there were more CD56^dim^ subsets that produced cytokines like IFNγ than CD56^bright^ NK cells (Table 8). Another study has suggested that NK cells undergo a continuum of differentiation from CD56^bright^ to CD56^dim^ NK cells and has identified a CD56^dim^ CD94^high^ subset [30]. We have also identified several intermediate CD16^+/-^ CD11b^+^ CD94^+^ CD62L^+^ subsets that were expressed by a higher frequency of CD56^bright^ NK cells in both adults and children (Table 8). Due to low sample size, the 3 pediatric groups were pooled together when contrasting adults and children. Therefore, in order to supplement our treeplot analysis and determine differences between all four age groups, we also analyzed combinatorial subsets using SPADE. However, we were unable to include all individuals in each of the age groups in some of the SPADE plots. Although we experienced some technical limitations, qualitative assessment of phenotypic differences with SPADE is nevertheless useful and valid.

When quantifying NK cell receptor diversity, inhibitory receptors were found to have the highest overall diversity, while cytokines and effector molecules had the lowest (Fig 6). Given the highly polymorphic nature of KIRs [71], their high diversity is not unexpected. Furthermore, adults had the lowest developmental marker diversity, which could possibly be due to increased maturation of NK cells with age. It has been previously proposed that >30000 unique subsets exist in healthy adults, contributing to their NK cell diversity [41]. While we used the same approach as Horowitz *et al*. to calculate diversity indices, our values were much lower due to our inability to simultaneously examine all 45 parameters in our panels using flow cytometry. However, unlike this study, flow cytometry allowed the calculation of MFI and clearly distinguished CD56^bright^ and CD56^dim^ NK cells, both of which have provided valuable insight.

Having quantified NK cell diversity, the question of NK cell repertoire stability arises. A prior study examined the NK cell repertoire stability in 12 healthy adults and found that the phenotype was stable up to 6 months but could be rapidly modified on cytokine stimulation [33]. Another study has looked at the changes in NK cell receptor expression in 3 individuals over a maximum period of 7 months up to 3 years [39]. In this study, NK cells were evaluated at 3–7 timepoints at intervals ranging from 1 week to 2 years. However, this study discovered that there was significant variation in NK cell receptors over time. While these represent non-overlapping time samples, they may reflect some inherent plasticity within the NK cell population. The study with the 12 healthy adults, however, reported specifically that the participants enrolled in their study did not acquire any chronic infections during the 6-month period, which is likely responsible for the stability of the NK cell repertoire [33]. Thus, these prior contributions demonstrate that while NK cell phenotype may remain stable for a short period, they do not lose the ability to harness their inherent plasticity.

In addition to determining differences by age, we also evaluated overall variation by sex and ethnicity. A recent study proposed that NK cell cytotoxicity is influenced by gender in the elderly [81]. Although, we did not measure cytotoxicity, few NK cell receptors differed in frequency between the sexes and among distinct ethnicities with Hispanics generally having the highest or lowest frequencies of these. Genetic associations with disease are often specific to race or ethnicity [82–85]. Therefore, it is crucial to understand how NK cell phenotype differs by ethnicity in healthy individuals. However, our study only focused on the protein expression and did not address any genotype-phenotype correlation. Nonetheless, since the individual expression of most NK cell markers did dot differ, our data can thus be applied to a variety of ethnic backgrounds. Apart from age, sex and ethnicity, allergies have also been shown to influence the NK cell phenotype [86]. Given the prevalence of food allergy among children in the United States (1:13), we did not exclude children with food allergy from our healthy cohort [87, 88] and in fact recruited some pediatric volunteers from an allergy clinic. However, with the exception of TNFα, which was lower, none of the other NK cell markers in our panels differed in children with food allergy (S4 Fig). Lastly, we cannot rule out the impact of physical exercise, stress or depressive disorder on NK cell counts and function [89, 90].

We found that there was greater disparity between adults and children on the whole, rather than among the three pediatric groups, when comparing individual expression of NK cell markers in our panels. While the developing immune system undergoes the greatest changes in the first few years of life, it does not reach immunological maturity until adolescence [91]. Moreover, viral infections are known to shape the NK cell repertoire and they occur progressively throughout childhood given the initial exposures to these ubiquitous agents. hCMV infection causes expansion of a specific NKG2C^+^ NK cell subset [20]. Although we do not have access to the CMV status of our cohort, our data showed that the MFI of NKG2C increased in children above ten. Several studies have examined the NK cell phenotype in children below five, but our knowledge of NK cells in children above that age is limited. Only one study has determined the expression of receptors on both CD56^bright^ and CD56^dim^ NK cells in healthy children over five and distinguished them from adults [37]. However, this study provides limited phenotyping and our extended comprehensive panels and age categories therefore fills a gap in the field of NK cell biology.

Small scale clinical studies that have explored phenotypic differences in a patient have been limited to one healthy control [73]. The diversity of NK phenotype we have demonstrated in healthy controls suggests that the use of only one healthy control as a comparator is not appropriate. Furthermore, extended phenotyping in diseases involving NK cells such as NK cell deficiencies, HIV and cancer are largely missing and warrant further investigation as well as context within knowledge of a presumably healthy population. Thus, the NK cell subsets and normative ranges identified in the present work will hopefully aid in interpreting clinical observations of NK cells in the pediatric population.

## Supporting information

S1 Fig Gating strategy. (PDF)

S2 Fig Variation in total NK cell frequencies and CD56^bright^ and CD56^dim^ subsets with age. (A) Percentage of NK cells as a proportion of total CD45^hi^ lymphocytes across 4 age groups. (B) Percentages of CD56^bright^ and CD56^dim^ cells as a proportion of total NK cells across 4 age groups. Top row represents CD56^dim^ NK cells and bottom row is CD56^bright^ NK cells. Each data point is an average of 3 repeats per donor. All data shown is mean ± interquartile range. 5–10 y.o. (n = 15) (red); 11–15 y.o. (n = 15) (green); 16–20 y.o. (n = 15) (blue) and adults (n = 20) (black).
(PDF)

S3 Fig MFI of NKG2C increases with age. Median fluorescent intensity (MFI) of NK cells with positive expression of NKG2C among 4 age groups. All data shown is mean ± interquartile range. Each data point represents a donor; 5–10 y.o. (n = 15) (red); 11–15 y.o. (n = 15) (green); 16–20 y.o. (n = 15) (blue) and adults (n = 20) (black).
(PDF)

S4 Fig Intracellular production of TNFα is reduced in children with food allergy. Percentage of NK cells with positive expression of TNFα post-stimulation based on food allergy status. Each data point represents a donor; No (n = 24); Yes (n = 15); and Unknown (n = 6). All data shown is mean ± S.D. compared by Kruskal-Wallis and post-hoc comparison with Bonferroni.
(PDF)

S1 Table Patient cohort. (PDF)

S2 Table List of antibodies. (PDF)

S3 Table Percent and fold change in frequency and MFI of most relevant markers/ subsets in unstimulated NK cells. (A) Each marker that is denoted by arrows in Table 9 and has a percent change of >10%, fold change >0.4 and significantly differed at p<0.01 from adults is highlighted in red. (B) Each combinatorial subset that has a percent change of >10%, fold change >0.4 and significantly differed at p<0.01 from adults is stated.
(PDF)

S4 Table Percent and fold change in frequency and MFI of most relevant markers/ subsets in stimulated NK cells. Each marker that is denoted by arrows in Table 10 and significantly differed at p<0.01 from adults is highlighted in red.
(PDF)

S1 Dataset Total NK cell frequency and MFI. (XLSX)

S2 Dataset CD56^bright^ and CD56^dim^ NK cell frequency, MFI and significantly different p-values. (XLSX)

S3 Dataset Frequency of NK cell combinatorial subsets. (XLSX)
