# Supplementary figures and images for: High-resolution phenotyping identifies NK cell subsets that distinguish healthy children from adults
Source: PLoS One. 2017 Aug 2;12(8):e0181134. doi: 10.1371/journal.pone.0181134 (PMC5540415; doi:10.1371/journal.pone.0181134)

[Ungated] FSC-A / SSC-A

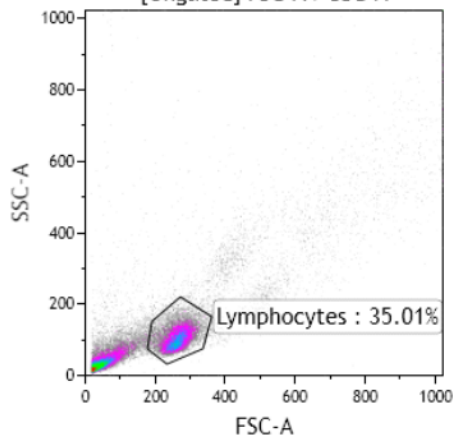

[Lymphocytes] SSC-H / SSC-W

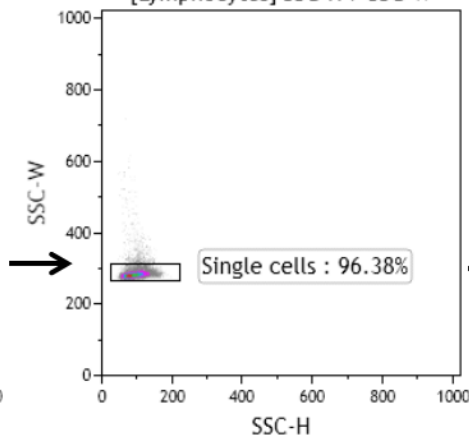

[Single cells] FSC-H / FSC-W

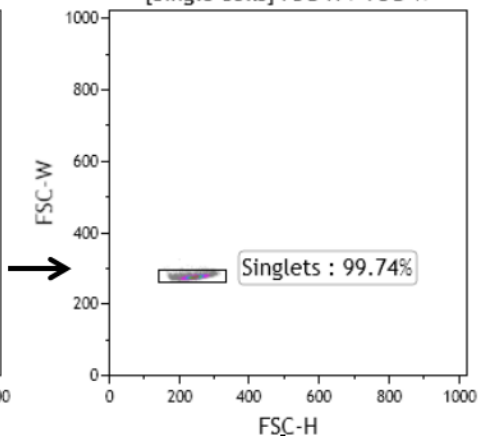

[Singlets] CD45 / SSC-A

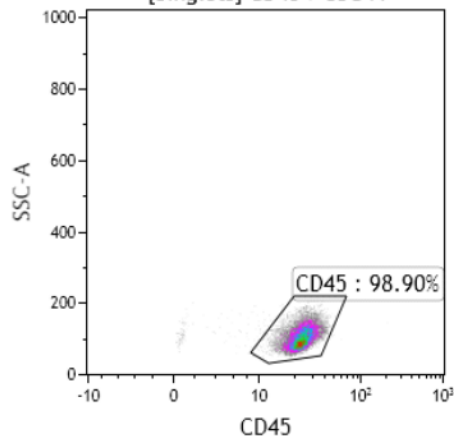

[CD45] CD3 / CD56

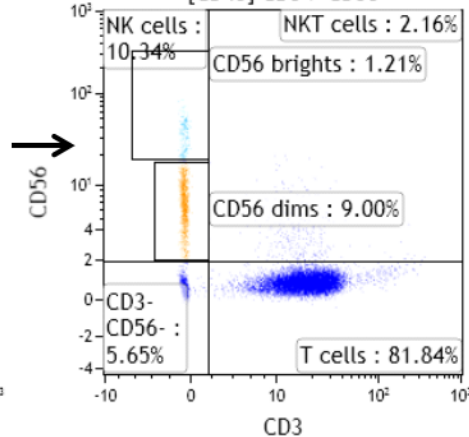

Supplement: S1 Fig — (PDF) [file pone.0181134.s001.pdf]

A

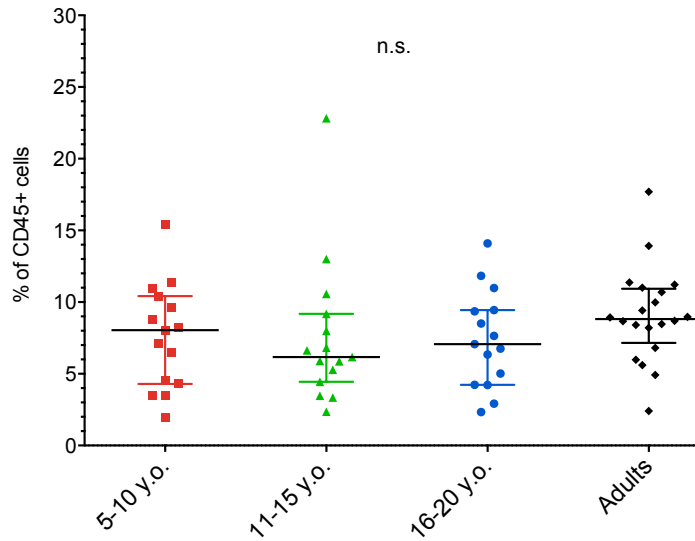

B

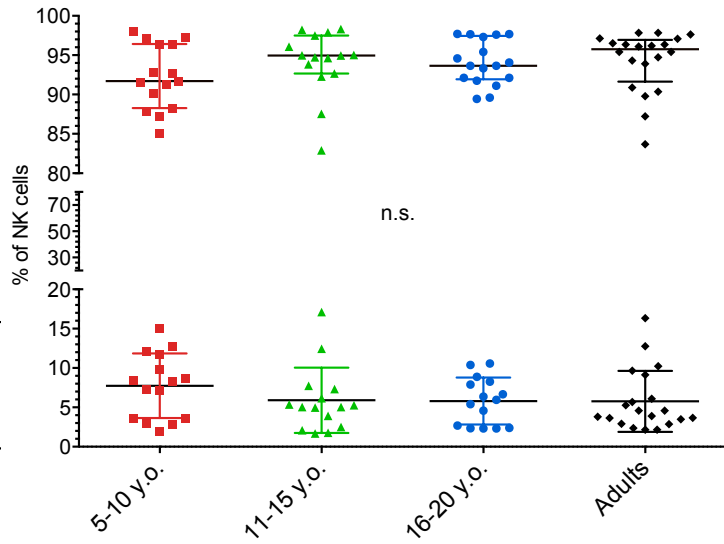

Supplement: S2 Fig — (A) Percentage of NK cells as a proportion of total CD45hi lymphocytes across 4 age groups. (B) Percentages of CD56bright and CD56dim cells as a proportion of total NK cells across 4 age groups. Top row represents CD56dim NK cells and bottom row is CD56bright NK cells. Each data point is an average of 3 repeats per donor. All data shown is mean ± interquartile range. 5–10 y.o. (n = 15) (red); 11–15 y.o. (n = 15) (green); 16–20 y.o. (n = 15) (blue) and adults (n = 20) (black). (PDF) [file pone.0181134.s002.pdf]

MFI of NKG2C positive NK cells

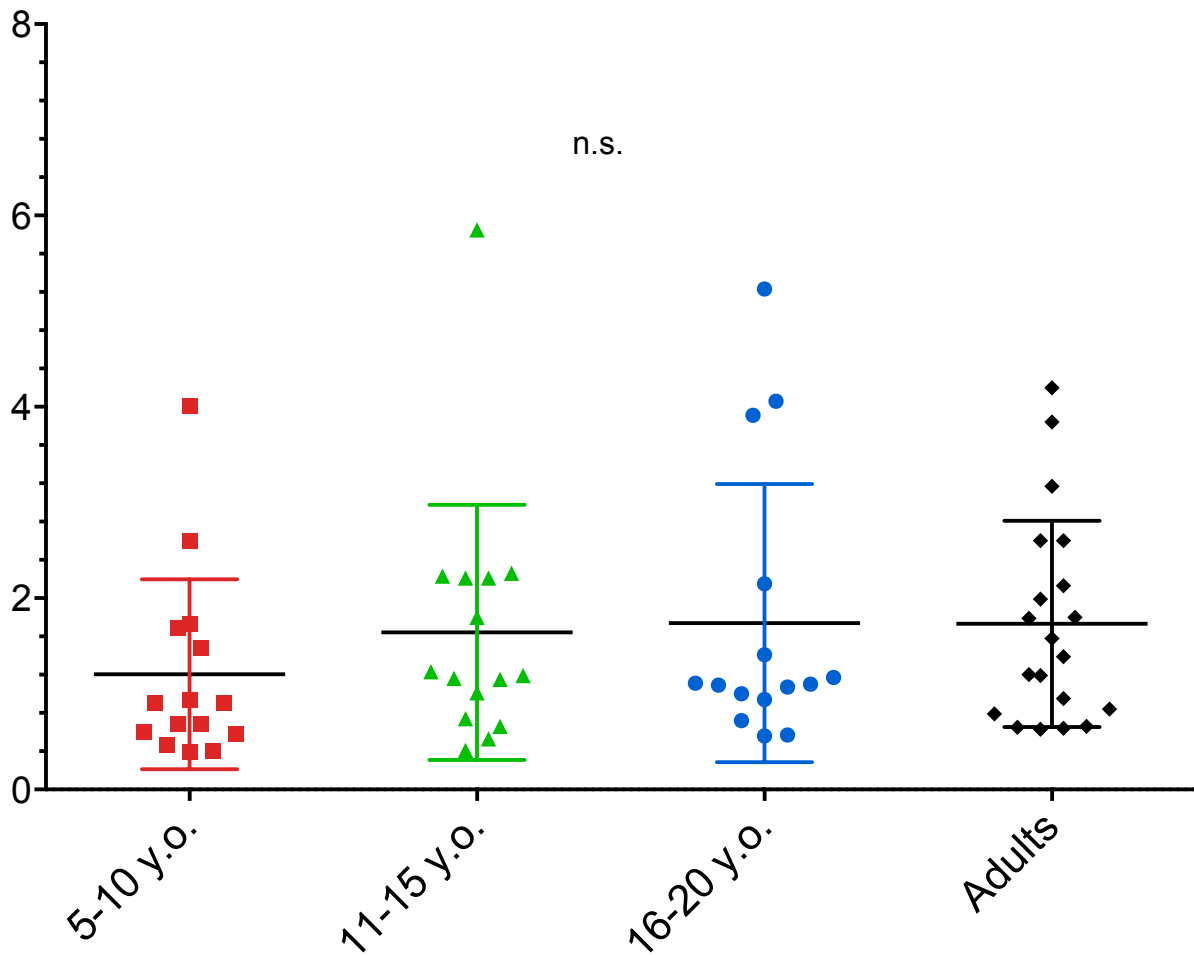

Supplement: S3 Fig — Median fluorescent intensity (MFI) of NK cells with positive expression of NKG2C among 4 age groups. All data shown is mean ± interquartile range. Each data point represents a donor; 5–10 y.o. (n = 15) (red); 11–15 y.o. (n = 15) (green); 16–20 y.o. (n = 15) (blue) and adults (n = 20) (black). (PDF) [file pone.0181134.s003.pdf]

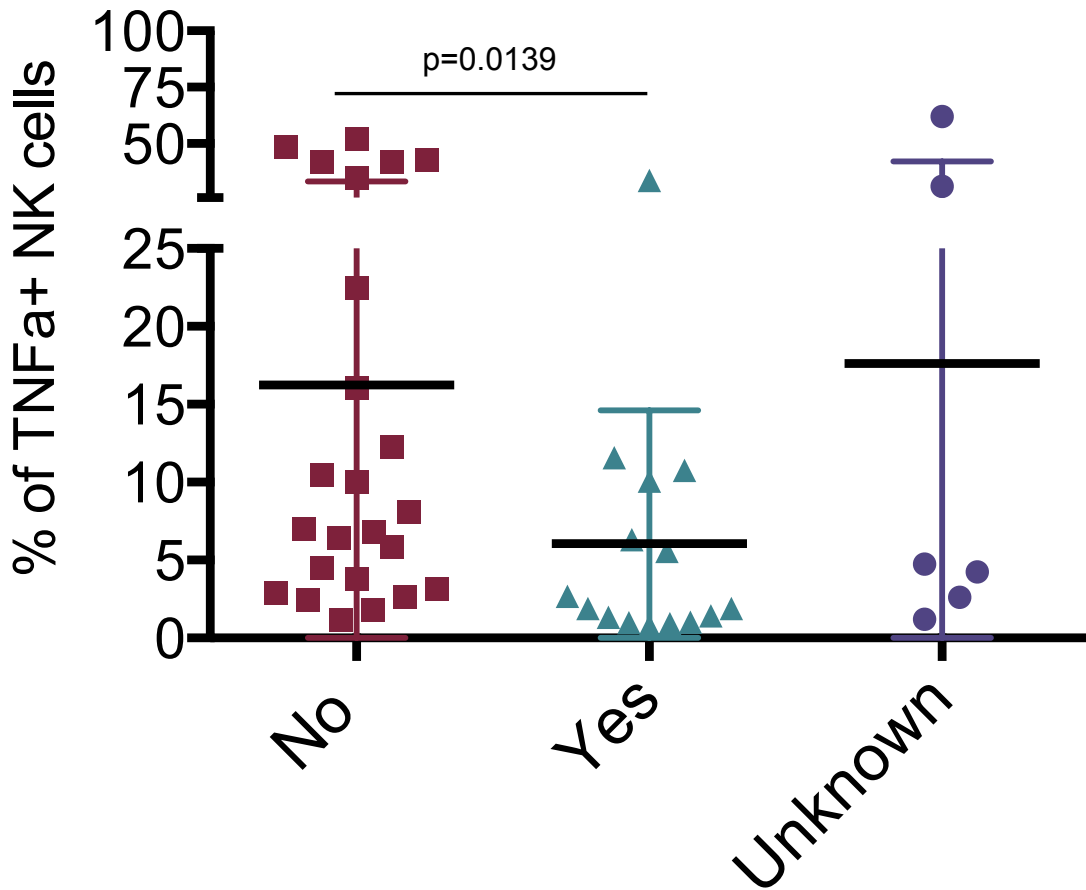

Supplement: S4 Fig — Percentage of NK cells with positive expression of TNFα post-stimulation based on food allergy status. Each data point represents a donor; No (n = 24); Yes (n = 15); and Unknown (n = 6). All data shown is mean ± S.D. compared by Kruskal-Wallis and post-hoc comparison with Bonferroni. (PDF) [file pone.0181134.s004.pdf]
