# Supplementary material for: High-resolution phenotyping identifies NK cell subsets that distinguish healthy children from adults
Source: PLoS One. 2017 Aug 2;12(8):e0181134. doi: 10.1371/journal.pone.0181134 (PMC5540415; doi:10.1371/journal.pone.0181134)
Supplement: S1 Table — (PDF) [file pone.0181134.s005.pdf]

		Sex	Age	Ethnicity
1	5-10 y.o.	M	5	White (non-Hispanic)
2		F	5	White (non-Hispanic)
3		M	5	White (non-Hispanic)
4		M	6	Hispanic
5		M	6	White (non-Hispanic)
6		M	6	White (non-Hispanic)
7		F	7	White (non-Hispanic)
8		M	7	White (non-Hispanic)
9		F	8	Asian
10		F	8	White (non-Hispanic)
11		F	8	White (non-Hispanic)
12		M	9	Hispanic
13		F	9	Hispanic
14		M	9	Black
15		M	10	Black
16	11-15 y.o.	M	11	White (non-Hispanic)
17		M	11	Hispanic
18		F	11	Black
19		M	12	Hispanic
20		M	12	Black
21		M	12	Black
22		F	13	White (non-Hispanic)
23		F	13	Asian
24		M	13	Hispanic
25		F	14	Hispanic
26		F	14	White (non-Hispanic)
27		M	14	White (non-Hispanic)
28		F	15	Hispanic
29		F	15	Hispanic
30		F	15	Black
31	16-20 y.o.	M	16	Unknown
32		F	16	Hispanic
33		M	16	Hispanic
34		F	16	Hispanic
35		F	16	Black
36		F	17	Black
37		F	18	Black
38		M	18	Asian
39		F	18	Hispanic
40		F	18	Black
41		F	18	Black
42		F	19	White (non-Hispanic)
43		M	19	Black
44		F	19	Asian
45		F	20	Asian
46	Adults	M	22	White (non-Hispanic)
47		M	22	Asian
48		F	23	White (non-Hispanic)
49		F	24	White (non-Hispanic)
50		M	25	White (non-Hispanic)
51		M	25	Black
52		F	26	Asian
53		M	29	Asian
54		M	29	Asian
55		M	30	Asian
56		M	31	Asian
57		F	33	White (non-Hispanic)
58		M	35	Hispanic
59		F	36	Asian
60		F	36	White (non-Hispanic)
61		M	42	Asian
62		F	50	White (non-Hispanic)
63		F	52	Hispanic
64		F	54	White (non-Hispanic)
65		F	60	White (non-Hispanic)
