# Supplementary material for: High-resolution phenotyping identifies NK cell subsets that distinguish healthy children from adults
Source: PLoS One. 2017 Aug 2;12(8):e0181134. doi: 10.1371/journal.pone.0181134 (PMC5540415; doi:10.1371/journal.pone.0181134)
Supplement: S2 Table — (PDF) [file pone.0181134.s006.pdf]

Antibody	Clone	Fluorochrome	Catalog number	Antibody Source	Host	Antibody dilution
CD45	J.33	Krome Orange	A96416	Beckman Coulter	Mouse	1/34
NKp46	9E2	Pacific Blue	331912	BioLegend	Mouse	4/85
CD56	HCD56	Brilliant Violet 605	318334	BioLegend	Mouse	1/85
CD16	3G8	Brilliant Violet 650	302041	BioLegend	Mouse	1/340
CD3	OKT3	Brilliant Violet 711	317328	BioLegend	Mouse	1/170
CD8α	RPA-T8	Brilliant Violet 785	301046	BioLegend	Mouse	1/85
DNAM-1	11A8	FITC	338304	BioLegend	Mouse	1/17
NKp44	Z231	PE	IM3710	Beckman Coulter	Mouse	1/17
NKG2D	1D11	PE-Cy7	320811	BioLegend	Mouse	1/68
CD69	FN50	PE-CF594	562617	BD Biosciences	Mouse	1/68
NKp30	P30-15	APC	325209	BioLegend	Mouse	1/85
CD16	B73.1	APC Cy7	561306	BD Biosciences	Mouse	1/17
CD25	B1.49.9	APC-Alexa Fluor 700	A86356	Beckman Coulter	Mouse	1/85
CD2	39C1.5	Pacific Blue	B09685	Beckman Coulter	Rat	1/34
CD244	2-69	FITC	550815	BD Biosciences	Mouse	1/17
CD11c	BU15	PerCP Cy5.5	B19719	Beckman Coulter	Mouse	1/34
CD28	L293	PE	348047	BD Biosciences	Mouse	1/17
CD11a	HI111	PE-Cy7	561387	BD Biosciences	Mouse	1/85
CD54	HA58	PE-Cy5	555512	BD Biosciences	Mouse	1/17
CD11b	ICRF44	PE-CF594	562399	BD Biosciences	Mouse	1/85
CD18	6.7	APC	551060	BD Biosciences	Mouse	1/17
CD158e	DX9	Brilliant Violet 421	312714	BioLegend	Mouse	3/64
CD158b	DX27	FITC	312604	BioLegend	Mouse	1/32
CD94	HP-3D9	PerCP Cy5.5	562361	BD Biosciences	Mouse	1/80
NKG2C	134591	PE	FAB138P	R&D Systems	Mouse	1/32
CD158a/h/g	HP-MA4	PE-Cy7	25-1589-42	Affymetrix eBioscience	Mouse	1/32
KIR2DS4	179315	APC	FAB1847A	R&D Systems	Mouse	1/40
KLRG1	REA226	APC-Vio770	130-103-642	Miltenyi Biotec	Recombinant	3/64
NKG2A	131411	Alexa Fluor 700	FAB1059N	R&D Systems	Mouse	1/80
CD57	NC1	Pacific Blue	A74779	Beckman Coulter	Mouse	1/160
CD62L	DREG-56	Brilliant Violet 650	304831	BioLegend	Mouse	1/160
CD127	A019D5	Brilliant Violet 785	351330	BioLegend	Mouse	1/64
IL-15Rα	151303	Alexa Fluor 488	FAB1471G	R&D Systems	Mouse	1/80
CD122	TU27	PE	339006	BioLegend	Mouse	1/80
CD117	104D2D1	PE-Cy7	IM3698	Beckman Coulter	Mouse	1/16
CD27	O323	PE-Cy5	15-0279-42	Affymetrix eBioscience	Mouse	1/64
CD16	3G8	PE-CF594	562293	BD Biosciences	Mouse	1/320
CD94	DX22	APC	305508	BioLegend	Mouse	1/80
CD11b	Bear1	APC-Alexa Fluor 750	A97052	Beckman Coulter	Mouse	1/32
Perforin	B-D48	Brilliant Violet 421	353307	BioLegend	Mouse	1/80
TNFα	MAb11	Brilliant Violet 650	502938	BioLegend	Mouse	1/32
Perforin	δG9	FITC	556577	BD Pharmingen	Mouse	1/20
IL-5	JES1-39D10	PE	500904	BioLegend	Rat	1/32
IL-10	JES3-9D7	PE-Cy7	501420	BioLegend	Rat	1/32
CD107a	eBioH4A3	PE-Cy5	15-1079-42	Affymetrix eBioscience	Mouse	1/64
Granzyme B	GB11	PE Texas-Red	GRB17	ThermoFisher Scientific	Mouse	1/64
IL-13	JES10-5A2	APC	501908	BioLegend	Rat	1/32
IFNγ	4S.B3	Alexa Fluor 700	502520	BioLegend	Mouse	1/32
