# Supplementary material for: High-resolution phenotyping identifies NK cell subsets that distinguish healthy children from adults
Source: PLoS One. 2017 Aug 2;12(8):e0181134. doi: 10.1371/journal.pone.0181134 (PMC5540415; doi:10.1371/journal.pone.0181134)
Supplement: S3 Table — (A) Each marker that is denoted by arrows in Table 9 and has a percent change of >10%, fold change >0.4 and significantly differed at p<0.01 from adults is highlighted in red. (B) Each combinatorial subset that has a percent change of >10%, fold change >0.4 and significantly differed at p<0.01 from adults is stated. (PDF) [file pone.0181134.s007.pdf]

Value	Subset	NK marker	Adults	5-10 y.o.		11-15 y.o.		16-20 y.o.	
**A**	**Percent and fold change in frequency and MFI of most relevant NK cell markers**								
				**Fold change**	**% Diff**	**Fold change**	**% Diff**	**Fold change**	**% Diff**
%	$CD56^{dim}$	**NKp46**	10.31%	0.84	8.65	0.98	10.08	0.51	5.30
		**CD62L**	16.90%	0.89	15.06	1.05	17.82	0.42	7.07
	$CD56^{bright}$	**Perf-D48**	57.71%	0.52	30.09	0.44	25.65	0.41	23.67
		**Perf-δG9**	46.19%	0.63	28.91	0.49	22.71	0.51	23.78
MFI	$CD56^{dim}$	**NKp46**	0.83	4.66		4.70		3.57	
		**CD69**	4.25	-0.45		-0.39		-0.32	
		**2B4**	2.6	3.31		3.11		3.21	
		**CD11c**	8.36	-0.41		-0.31		-0.01	
		**CD158a (2DL1)**	3.11	0.59		0.55		0.05	
		**CD62L**	4.81	0.36		0.48		0.16	
		**IFNγ**	6.24	-0.44		-0.44		-0.31	
		**CD107a**	5.56	0.67		0.27		0.61	
	$CD56^{bright}$	**NKp46**	2.13	2.55		2.48		2.16	
		**2B4**	2.32	3.10		2.80		2.46	
		**CD107a**	6.13	0.37		0.81		0.19	

Value	Subset	NK subset	Adults (Mean %)	Children (Mean %)	% Diff	Fold change
**B**	**Percent change in frequency of most relevant NK cell combinatorial subsets**					
%	$CD56^{dim}$	CD16 3G8+CD11a+CD11b+CD11c+CD18+CD54+CD244+CD2+	13.71	26.4	12.69	0.93
	$CD56^{bright}$	CD16 3G8+CD11a+CD11b+CD11c+CD18+CD54+CD244+CD2+	14.95	36.97	22.02	1.47
		Perf D48-Perf δG9-Granzyme-CD107a-IFNγ-IL-5-IL-10-IL-13-	25.37	11.31	-14.06	-0.55
		Perf D48+Perf δG9+Granzyme-CD107a-IFNγ-IL-5-IL-10-IL-13-	4.7	23.98	19.28	4.10
