# Supplementary material for: High-resolution phenotyping identifies NK cell subsets that distinguish healthy children from adults
Source: PLoS One. 2017 Aug 2;12(8):e0181134. doi: 10.1371/journal.pone.0181134 (PMC5540415; doi:10.1371/journal.pone.0181134)
Supplement: S4 Table — Each marker that is denoted by arrows in Table 10 and significantly differed at p<0.01 from adults is highlighted in red. (PDF) [file pone.0181134.s008.pdf]

Value	Subset	NK marker	Adults (Mean)		5-10 y.o.		11-15 y.o.		16-20 y.o.	
			Fold change	% Diff	Fold change	% Diff	Fold change	% Diff	Fold change	% Diff
%	CD56dim	**CD69**	1.06	31.19	0.89	19.03	0.79	20.82	1.00	20.19
	CD56bright	**CD69**	1.36	41.07	1.87	19.38	2.16	29.21	2.41	38.14
MFI	CD56dim	**NKp46**	-0.84		-0.01		-0.03			-0.13
		**CD69**	0.43		0.34		0.49			0.52
		**IFNγ**	0.46		0.48		0.56			0.43
	CD56bright	**NKp46**	-0.69		-0.23		-0.24			-0.38
		**IFNγ**	0.37		0.65		0.50			0.45
		**CD107a**	0.03		0.38		-0.12			0.63
